# Uncovering the non-histone interactome of the BRPF1 bromodomain using site-specific azide-acetyllysine photochemistry

**DOI:** 10.1016/j.jbc.2023.105551

**Published:** 2023-12-10

**Authors:** Soumen Barman, Jyotirmayee Padhan, Babu Sudhamalla

**Affiliations:** Department of Biological Sciences, Indian Institute of Science Education and Research Kolkata, Mohanpur, West Bengal, India

**Keywords:** BRPF1, bromodomain, PTM, histones, acetyllysine, photo-cross-linking

## Abstract

Bromodomain-PHD finger protein 1 (BRPF1) belongs to the BRPF family of bromodomain-containing proteins. Bromodomains are exclusive reader modules that recognize and bind acetylated histones and non-histone transcription factors to regulate gene expression. The biological functions of acetylated histone recognition by BRPF1 bromodomain are well characterized; however, the function of BRPF1 regulation *via* non-histone acetylation is still unexplored. Therefore, identifying the non-histone interactome of BRPF1 is pivotal in deciphering its role in diverse cellular processes, including its misregulation in diseases like cancer. Herein, we identified the non-histone interacting partners of BRPF1 utilizing a protein engineering-based approach. We site-specifically introduced the unnatural photo-cross-linkable amino acid 4-azido-L-phenylalanine into the bromodomain of BRPF1 without altering its ability to recognize acetylated histone proteins. Upon photoirradiation, the engineered BRPF1 generates a reactive nitrene species, cross-linking interacting partners with spatio-temporal precision. We demonstrated the robust cross-linking efficiency of the engineered variant with reported histone ligands of BRPF1 and further used the variant reader to cross-link its interactome. We also characterized novel interacting partners by proteomics, suggesting roles for BRPF1 in diverse cellular processes. BRPF1 interaction with interleukin enhancer–binding factor 3, one of these novel interacting partners, was further validated by isothermal titration calorimetry and co-IP. Lastly, we used publicly available ChIP-seq and RNA-seq datasets to understand the colocalization of BRPF1 and interleukin enhancer–binding factor 3 in regulating gene expression in the context of hepatocellular carcinoma. Together, these results will be crucial for full understanding of the roles of BRPF1 in transcriptional regulation and in the design of small-molecule inhibitors for cancer treatment.

The bromodomain-PHD finger protein 1 (BRPF1) is a chromatin-associated scaffolding protein that forms a complex with the monocytic leukemic zinc-finger (MOZ) and MOZ-related factor histone acetyltransferase (HAT) (1, 2). The BRPF1–MOZ complex acetylates histone substrates and mediates transcription regulation (3). The role of the BRPF1–MOZ complex is critical in vertebrate development and diseases (4). Mutation of *Brpf1* leads to pattering defect of the skeleton in medaka fish and segmentation defect in zebrafish due to loss of *Hox* gene expression (3, 5). Inactivation of *Brpf1* in the forebrain of mice causes abnormal brain development, deregulated neuronal migration, and cell cycle progression causing a profound impact on hippocampus morphogenesis (6). Moreover, *BRPF1* mutation in humans causes intellectual disability, global developmental delay, and abnormalities in the spinal and central nervous system (7, 8, 9). In most of the cases of BRPF1 mutation, the functional outcome can be attributed to impaired control of histone acetylation (6, 7).

BRPF1 is a multidomain-containing protein that is recruited to the chromatin by orchestrating its various domain to interact with DNA and histones (10, 11). The domain organization of BRPF1 includes an N-terminal PZP domain (two PHD fingers connected by a zinc knuckle), a bromodomain, and a C-terminal PWWP (Pro-Trp-Trp-Pro) domain (12) (Fig. 1*A*). Recent biochemical and structural studies have shown that the PZP domain of BRPF1 associates with chromatin by recognizing the unmodified histone H3 tail and DNA (10, 11). The bromodomain of BRPF1 recognizes multiple acetyllysine residues on histone H4 and H3 (13, 14), whereas the PWWP domain recognizes H3K36me3 (15) (Fig. 1*B*). BRPF1 forms complex with MOZ/MOZ-related factor along with two additional subunits, hEAF6 and ING5, and acetylates several lysine residues on H3 and H4 tails (1, 16, 17, 18) (Fig. 1*C*).

**Figure 1:**
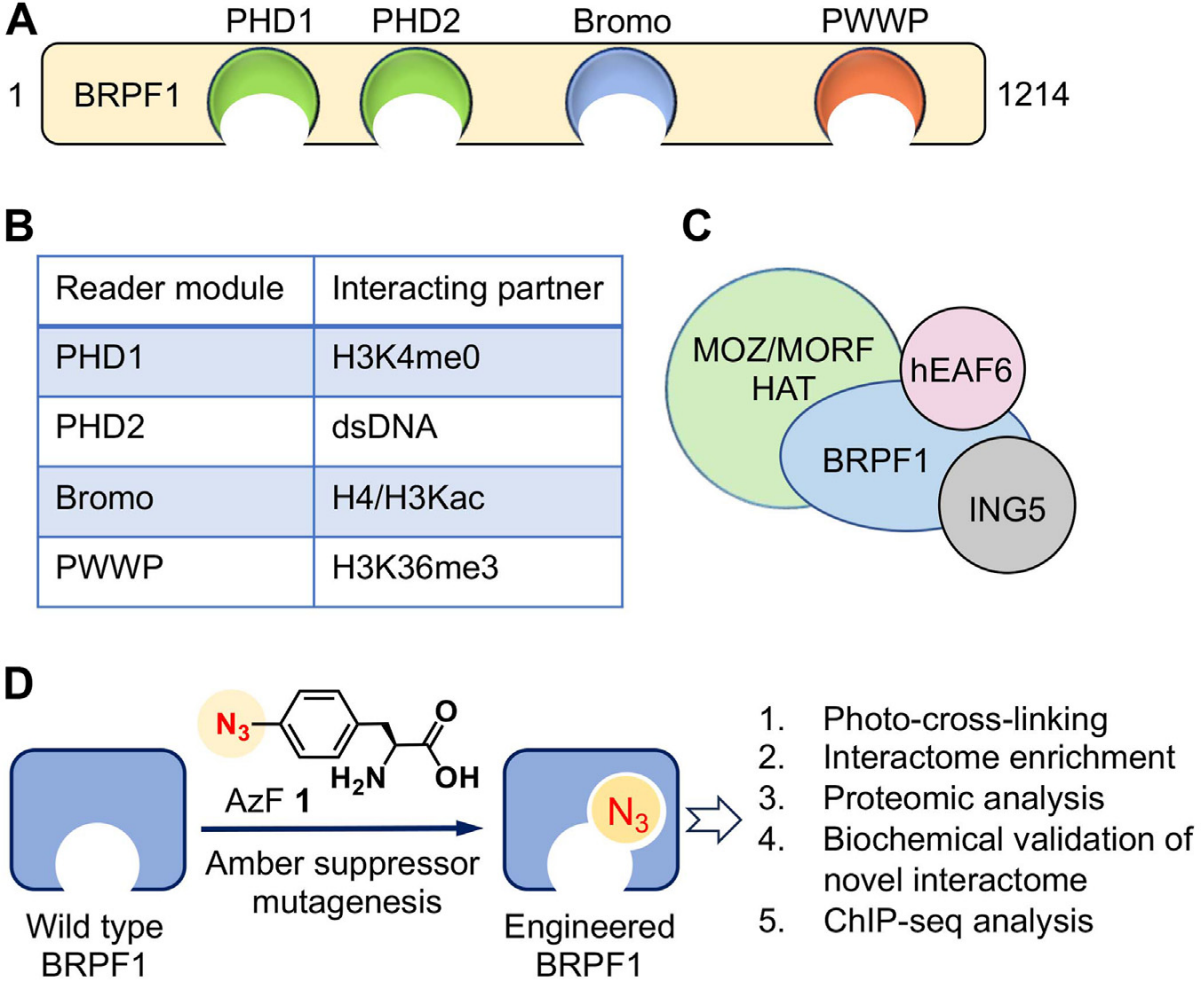
**Domain organization of BRPF1 and its bromodomain engineering**. *A*, BRPF1 is composed of tandem PHD fingers, a bromodomain, and a PWWP domain. *B*, different reader modules of BRPF1 binds to modified histones and DNA. *C*, BRPF1 forms a tetrameric complex with non-histone transcriptional regulators such as MOZ, MORF, hEAF6, and ING5. *D*, schematic representation of BRPF1 bromodomain engineering with photo-cross-linking amino acid 4-azido-L-phenylalanine (AzF 1) and profiling of its non-histone interactome. BRPF, bromodomain-PHD finger protein; MORF, MOZ-related factor; MOZ, monocytic leukemic zinc-finger.

The chromatin recruitment of bromodomain-containing proteins is not only driven by docking into acetylated histones, but their association with acetylated non-histone transcription factors also plays a crucial role in mediating their functions (19, 20, 21). An earlier study on the bromodomain-containing protein BRD4 has revealed its two bromodomain's role in controlling EMT gene expression in breast cancer (22). The first bromodomain of BRD4 binds to acetylated histones, while its second bromodomain binds to the transcription factor twist acetylated at two lysine residues (K73 and K76) that mimics histone sequence (22). This twist–BRD4 interaction is essential for expressing EMT-related genes and promotes tumorigenicity in breast cancer (22). Another recent study identified that the bromodomains of p300 and CBP bind to E2F1, which becomes acetylated in response to DNA damage (23). The HATs p300/CBP are recruited to the chromatin by binding acetylated E2F1 to further acetylate histone H3 at K14 and K56 positions to initiate DNA repair (23). In another context, the dual bromodomain of TAF1 binds to the AML-ETO fusion transcription factor in an acetylation-dependent manner and helps in chromatin localization of AML-ETO in its target genes to promote leukemogenesis (24).

Identifying the non-histone interactome of epigenetic readers is challenging because the affinity of these interactions lies in the micromolar range, which cannot survive harsh treatment during methods like pulldown or immunoprecipitation (25). To overcome the above challenges, Islam and colleagues have developed an approach called interaction-based protein profiling (IBPP), whereby they engineered the epigenetic reader BRD4 site specifically to carry a photo-cross-linkable unnatural amino acid, 4-azido-L-phenylalanine (AzF) in the acetyllysine binding pocket of bromodomain (26). The photo-activable azido group forms reactive nitrene species upon UV irradiation, which can form strong covalent bond with nearby interacting partners. Using the IBPP approach, they identified some novel interacting partners of BRD4, emphasizing that the function of BRD4 expands far beyond transcription (26). Since then, many epigenetic readers like the bromodomain of ATAD2B, BRD1, TAF1, and BET family bromodomains have been successfully engineered to profile their novel interactome (27, 28, 29, 30). These photo-cross-linking unnatural amino acid engineering-based approaches are powerful methods for capturing transient protein–protein interactions with temporal precision (26, 27, 30, 31). Using these approaches, the vast non-histone interactome of epigenetic readers has been identified, which made us appreciate their role in diverse cellular functions besides transcription.

Like other bromodomain-containing proteins, BRPF1 too is involved in various cellular processes like skeletal development, vertebrate segmentation and patterning, brain development and even in cancer-like hepatocellular carcinoma (HCC) (12, 32). In HCC, it was shown that BRPF1 was among the most significantly upregulated bromodomain-containing protein and its expression negatively correlates with patients’ survival (32, 33). Moreover, inhibition of BRPF1 by CRISPR KO or treatment with BRPF1 bromodomain specific inhibitor GSK5959 inhibited HCC growth *in vivo* demonstrating that BRPF1s′ bromodomain specific functions are essential determinants of HCC tumorigenesis (32). The bromodomain of BRPF1 is well characterized, and its histone ligands have been identified by biophysical and biochemical approaches (13, 14). However, identifying the non-histone interactome of BRPF1 is crucial to get a deeper insight into the complex network of BRPF1-dependent functions. Therefore, in this study, we utilized the IBPP approach to engineer the bromodomain of BRPF1 with AzF 1 without compromising its ability to bind and cross-link its interacting partners. And the cross-linked novel interactome from the human proteome were further characterized by proteomics (Fig. 1*D*). As a proof-of-concept demonstration, we validated the interaction of BRPF1 and interleukin enhancer-binding factor 3 (ILF3), a novel non-histone interacting partner of BRPF1 and used publicly available ChIP-seq and RNA-seq datasets to understand the functional significance of this interaction in cellular context.

## Results and discussion

### Engineering the BRPF1 bromodomain with photo-cross-linking amino acid

**Figure 2:**
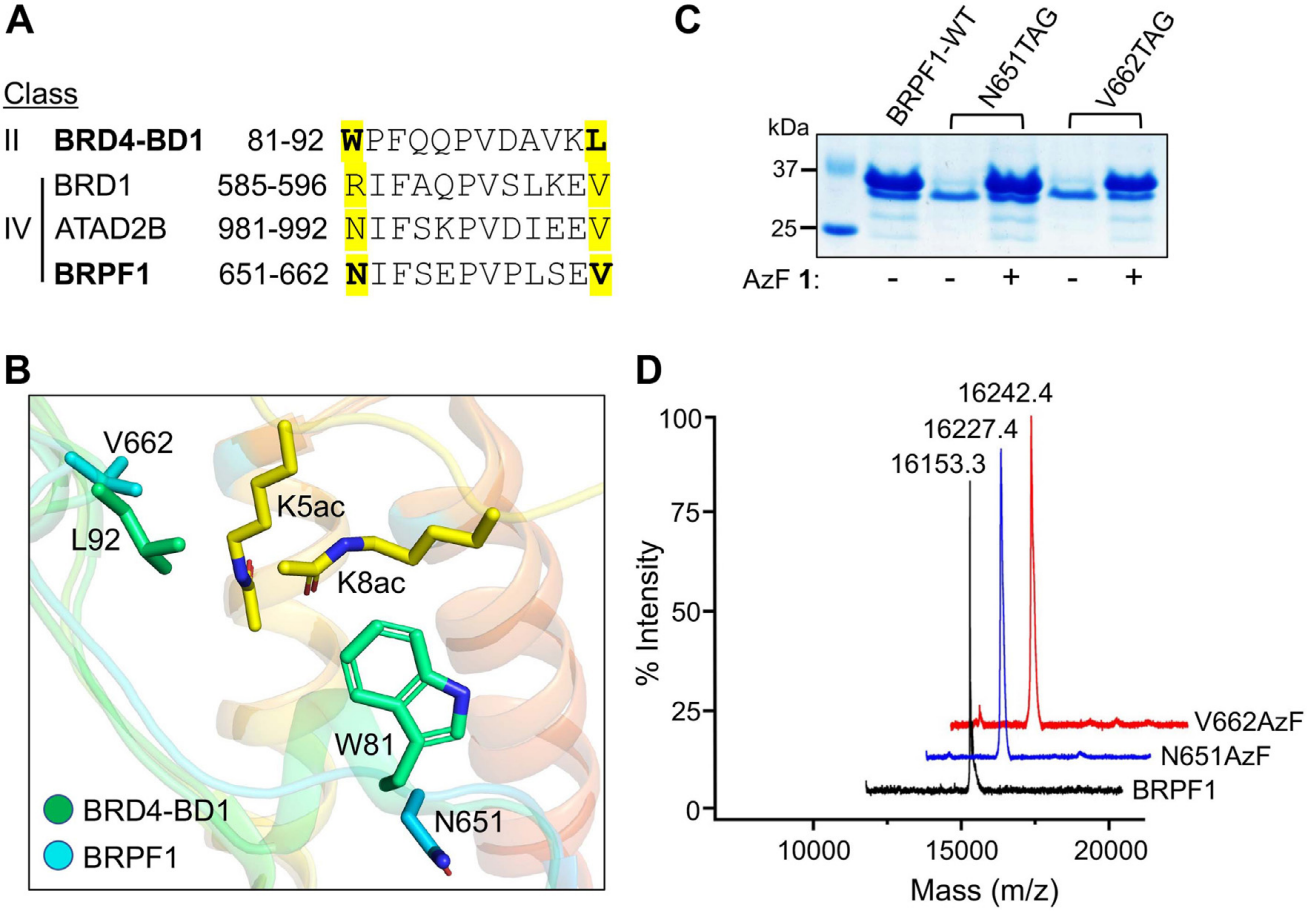
**Engineering the BRPF1 bromodomain with 4-azido-L-phynylalanine.***A*, aligned partial sequences of selected BET (BRD4-BD1) and non-BET (BRD1, ATAD2B, and BRPF1) family bromodomains. The BD1 residues (W81 and L92) of BRD4 and the corresponding residues (N651 and V662) in the BRPF1 bromodomain are shown in *bold* and highlighted in *yellow*. *B*, structural superposition of BRD4-BD1 (PDB entry 3UVW) and BRPF1 bromodomain (PDB entry 5FFW) showing the positions of selected residues. The selected BRPF1 bromodomain residues (N651 and V662) are subjected to AzF incorporation to develop photo-cross-linkable BRPF1. *C*, expression of WT BRPF1 bromodomain and its mutants in *Escherichia coli* cells in the absence or presence of AzF as judged by Coomassie blue staining. *D*, MALDI-MS spectrum confirming the N651AzF and V662AzF mutants bearing AzF. BRPF, bromodomain-PHD finger protein.

To engineer the BRPF1 bromodomain with photo-cross-linking amino acid AzF 1, we specifically targeted the amino acid residues in the hydrophobic cavity of the bromodomain (Fig. 2*A*). Our previous study demonstrated that certain amino acid residues in the hydrophobic cavity of the BET and non-BET bromodomains are amenable to the incorporation of photo-cross-linking amino acids without altering the binding affinity for its cognate ligand (26, 27, 28). For example, BET family protein BRD4 variants carrying W81AzF and L92AzF mutations in the first bromodomain can bind and cross-link with acetylated interacting partners. Sequence alignment and structural superimposition of the bromodomains reveal that (N651 and V662) of BRPF1 and (W81 and L92) of BRD4 occupy a similar position in the hydrophobic pocket (Fig. 2*A* and *B*). To develop a photo-activable analogs of BRPF1 bromodomain for profiling its transient non-histone interacting partners, we generated the mutants N651AzF and V662AzF *via* amber suppressor codon (TAG) mutagenesis using the evolved orthogonal *Methanococcus jannaschii* TyrRS-tRNA_CUA_^Tyr^ pair (34). The mutants were expressed in high yields and purified to high homogeneity (Figs. 2*C* and S1). MALDI-MS analysis confirmed the integrity of the intact proteins and the incorporation of AzF unit into mutant proteins (Fig. 2*D* and Table S4).

**Figure 3:**
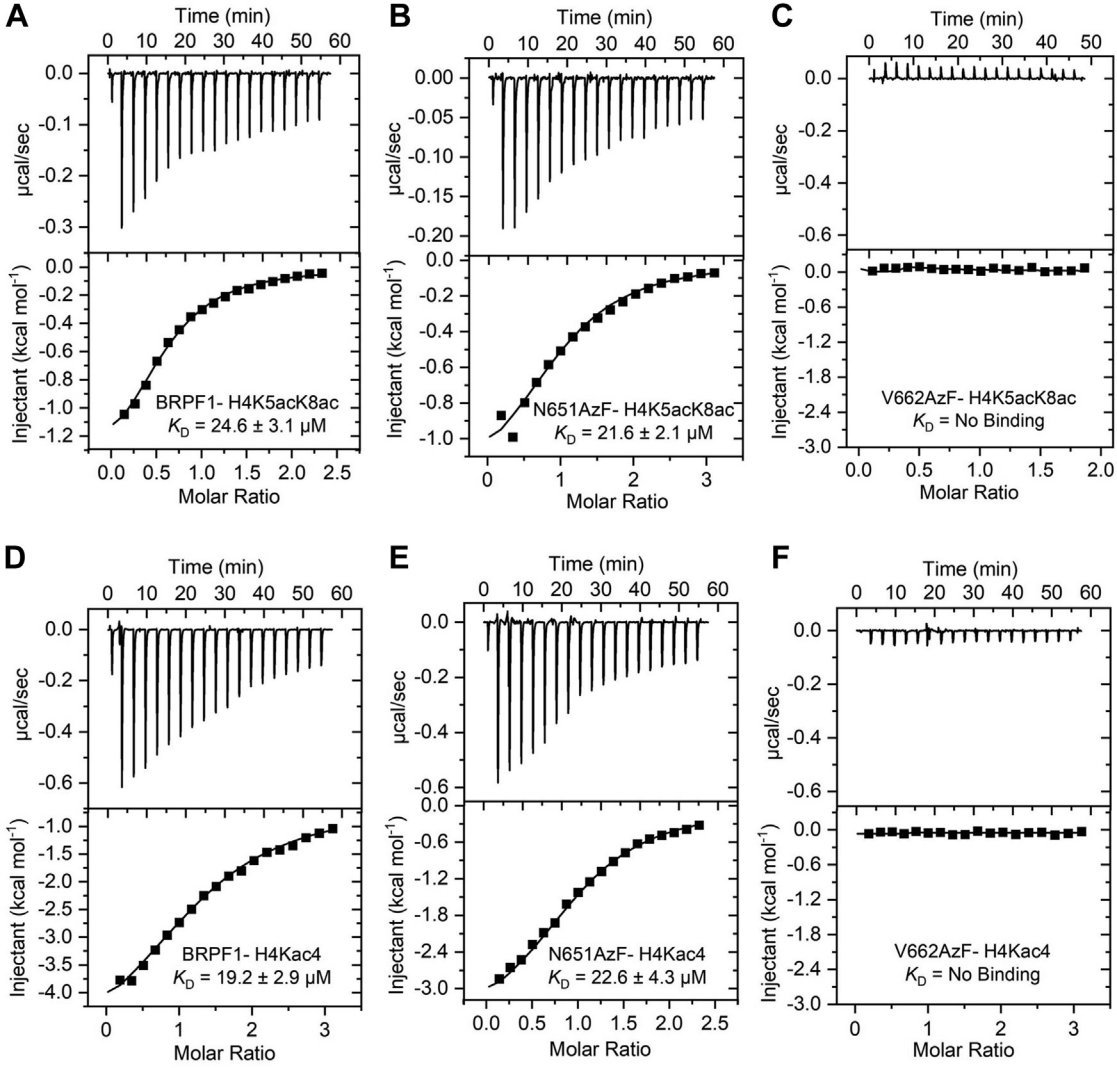
**ITC measurements of the interaction between bromodomain of BRPF1 and acetylated histone peptides.***A*–*F*, exothermic ITC plots for binding of the WT BRPF1 bromodomain and its AzF mutants (N651AzF and V662AzF) to the histone H4K5acK8ac peptide 2 and H4Kac4 (K5/K8/K12/K16) peptide 3. The calculated dissociation constants are indicated. AzF, 4-azido-L-phenylalanine; BRPF, bromodomain-PHD finger proteiny; ITC, isothermal titration calorimetry.

To examine the potential of the BRPF1 bromodomain mutants to bind acetylated histone substrates, we carried out isothermal titration calorimetry (ITC) binding assay with acetylated histone peptide 2 (Figs. S2 and S3 and Table S3). The WT BRPF1 bromodomain bound histone peptide 2 with a *K*_D_ value of 24.6 ± 3.1 μM (Fig. 3*A*), which is in good agreement with the reported value (14). Among the tested two mutants, only one mutant N651AzF showed a similar binding affinity toward peptide 2 with a *K*_D_ value of 21.6 ± 2.1 μM (Fig. 3*B*). A loss of binding was observed for the V662AzF mutant with the histone peptide 2 (Fig. 3*C*), and this is likely due to steric hindrance between the bromodomain binding pocket residue V662AzF and acetylated peptide 2. To further confirm the binding ability of mutant proteins, we synthesized a tetra-acetylated histone peptide 3 (Figs. S4 and S5 and Table S3) for the ITC binding studies. Our ITC binding results revealed that the WT BRPF1 bromodomain and its mutant N651AzF bound histone peptides 3 with similar efficiency with a *K*_D_ value of 19.2 ± 2.9 μM and 22.6 ± 4.3 μM, respectively (Fig. 3*D* and *E*). As expected, the V662AzF mutant failed to recognize the histone peptide 3 (Fig. 3*F*).

### MD simulations predict the possible orientation of photo-cross-linking amino acid in the acetyllysine binding pocket of BRPF1 bromodomain

**Figure 4:**
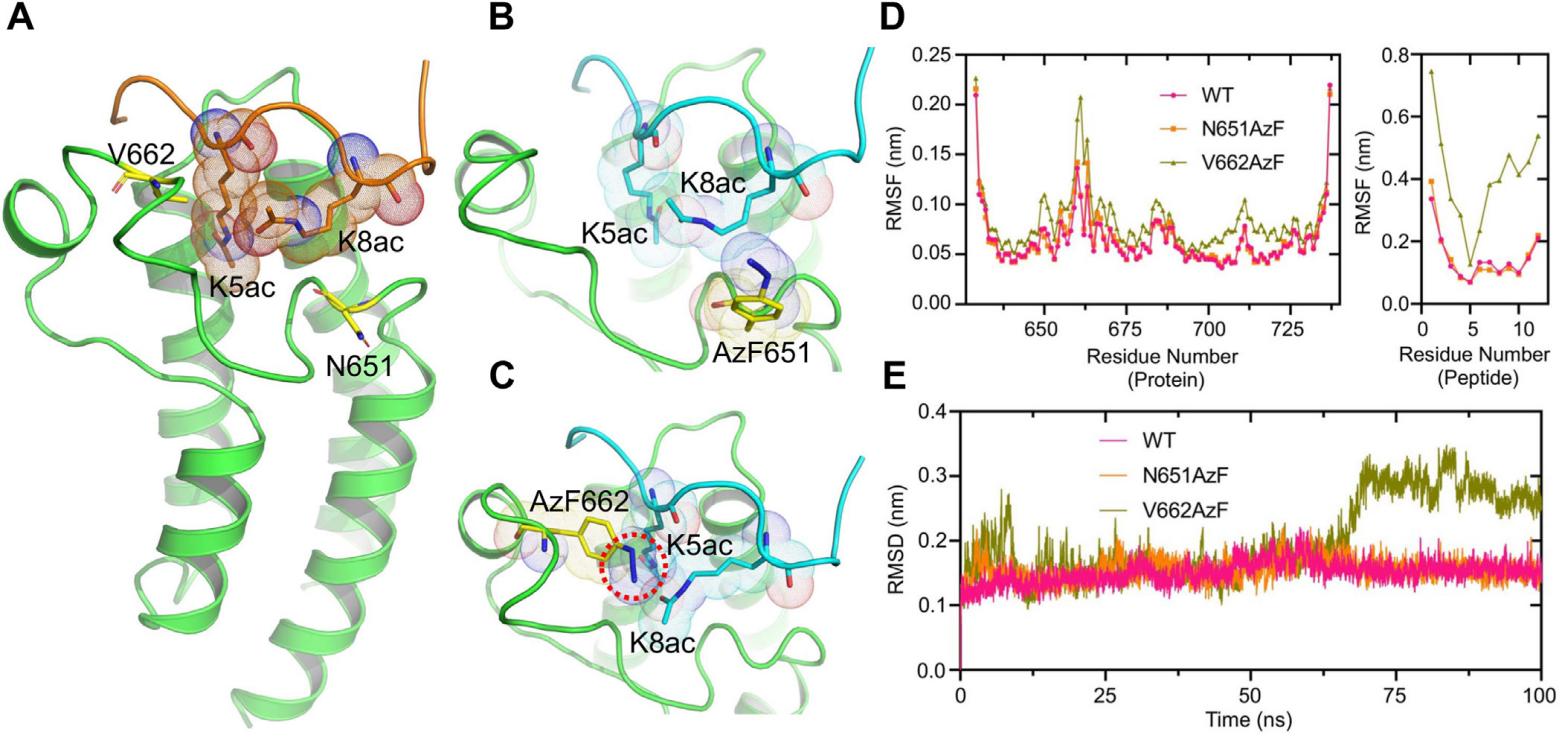
**Molecular modeling and MD simulation of BRPF1 bromodomain mutants.***A*, modeled structure of BRPF1 bromodomain with H4K5acK8ac peptide; *yellow stick* represents the selected residues for AzF mutation. *B* and *C*, 100th ns snapshot of BRPF1 mutants (AzF651 and AzF662) and H4K5acK8ac peptide complexes, respectively. The *dotted circle* highlights the *steric clash* between BRPF1-AzF662 and H4K5ac. *D*, Cα RMSF of the BRPF1 bromodomain and histone peptide *versus* residue number, respectively. *E*, Cα RMSD demonstrating the stability of the protein backbone during the MD simulation. AzF, 4-azido-L-phenylalanine; BRPF, bromodomain-PHD finger protein; MD, molecular dynamics.

To investigate the impact of V662AzF mutation on the acetyllysine recognition by the bromodomain, we created a modeled structure of BRPF1 bromodomain in complex with histone H4K5acK8ac peptide 2 (Fig. 4*A*). Next, we used molecular modeling to mutate the selected residues N651 and V662 in the bromodomain of BRPF1 with AzF. In addition, we used molecular dynamics (MD) simulation to predict the possible orientations of BRPF1-AzF mutants in the acetyllysine binding pocket of the bromodomain. MD snapshot analysis demonstrates that replacing N651 with AzF did not alter the acetyllysine binding (Fig. 4*B*). By contrast, V662AzF incorporation resulted in changing the orientation of the azide moiety and directly clashes with the acetyllysine, which prevents the ligand entry into the binding pocket (Fig. 4*C*). Moreover, analysis of rms fluctuation suggests that V662AzF mutant and histone peptide 2 displayed major fluctuations in the acetyllysine binding pocket of the bromodomain. Notably, N651AzF and histone peptide 2 showed similar WT fluctuations (Fig. 4*D*). Next, the RMSD analysis revealed that N651AzF and histone peptide 2 complexes were stable throughout the 100 ns simulation, but the V662AzF and histone peptide 2 complexes were significantly deviated from 50 ns to 100 ns simulation (Fig. 4*E*). Together, our ITC binding results are consistently supplemented by the MD simulation studies.

### Photo-cross-linking of engineered BRPF1 bromodomain with interacting partners

**Figure 5:**
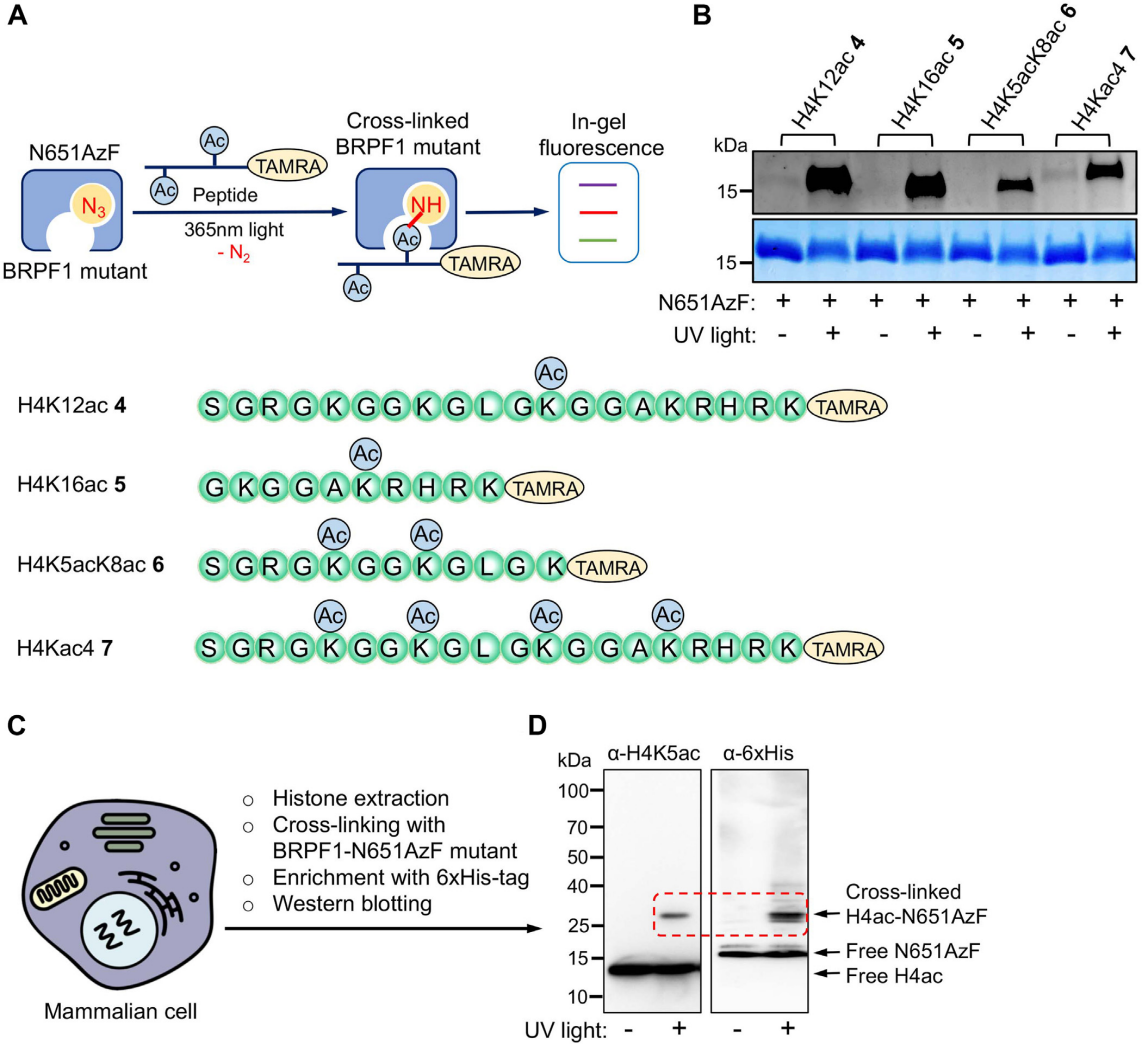
**Photo-cross-linking of engineered BRPF1 bromodomain with histone peptides and endogenous histones.***A*, schematic depicting the binding of engineered BRPF1 bromodomain to H4ac-TAMRA peptides 4 to 7, followed by cross-linking using 365 nm UV light. *B*, in-gel fluorescence showing crosslinking of BRPF1-N651AzF to TAMRA-labeled peptides 4 to 7. Coomassie staining of the same gel shows the presence of proteins in all of the samples. *C*, HEK293T cells were treated with deacetylase inhibitor (trichostatin A) for 24 h to generate the hyperacetylated histones. The extracted histones were incubated with BRPF1-N651AzF, followed by UV exposure and enriched with Ni-NTA bead *via* a His-tag on the protein (*D*) Western blot showing cross-linking of acetylated H4 (H4ac) with BRPF1-N651AzF mutant as confirmed by anti-H4K5ac and anti-6xHis antibodies. BRPF, bromodomain-PHD finger protein; TAMRA, tetramethylrhodamine.

Next, we selected the N651AzF mutant based on its binding ability and sought to examine its potential to cross-link with known interacting partners. We synthesized tetramethylrhodamine (TAMRA) attached H4K12ac peptide 4, H4K16ac peptide 5, H4K5acK8ac peptide 6, and H4Kac4 peptide 7 (Figs. S6–S13 and Table S3) and subjected them to cross-linking with N651AzF using 365 nm UV light (Fig. 5*A*). Subsequent enrichment with Ni-NTA beads and In-gel fluorescence revealed cross-linked bands of N651AzF mutant and labeled peptides 4 to 7 only upon exposure to UV light (Fig. 5*B*), demonstrating the N651AzF mutant capable of binding and cross-linking with different acetylated interacting partners.

To demonstrate the ability of N651AzF to bind and cross-link to endogenous histones, we generated hyperacetylated histones by treating the HEK293T cells with deacetylase inhibitor trichostatin A (Fig. 5*C*). Next, the isolated histones were incubated with N651AzF mutant, followed by cross-linking by irradiating with 365 nm UV light. The cross-linked proteins were enriched with Ni-NTA beads and analyzed by immunoblotting with anti-acetylated H4 and anti-6xHis antibodies showed higher molecular weight bands (Fig. 5*D*), suggesting the acetylated H4 underwent smooth cross-linking with N651AzF upon UV irradiation. No cross-linked product was observed when the samples were not exposed to UV light.

### Photo-cross-linking of the engineered BRPF1 bromodomain with the cellular proteome

**Figure 6:**
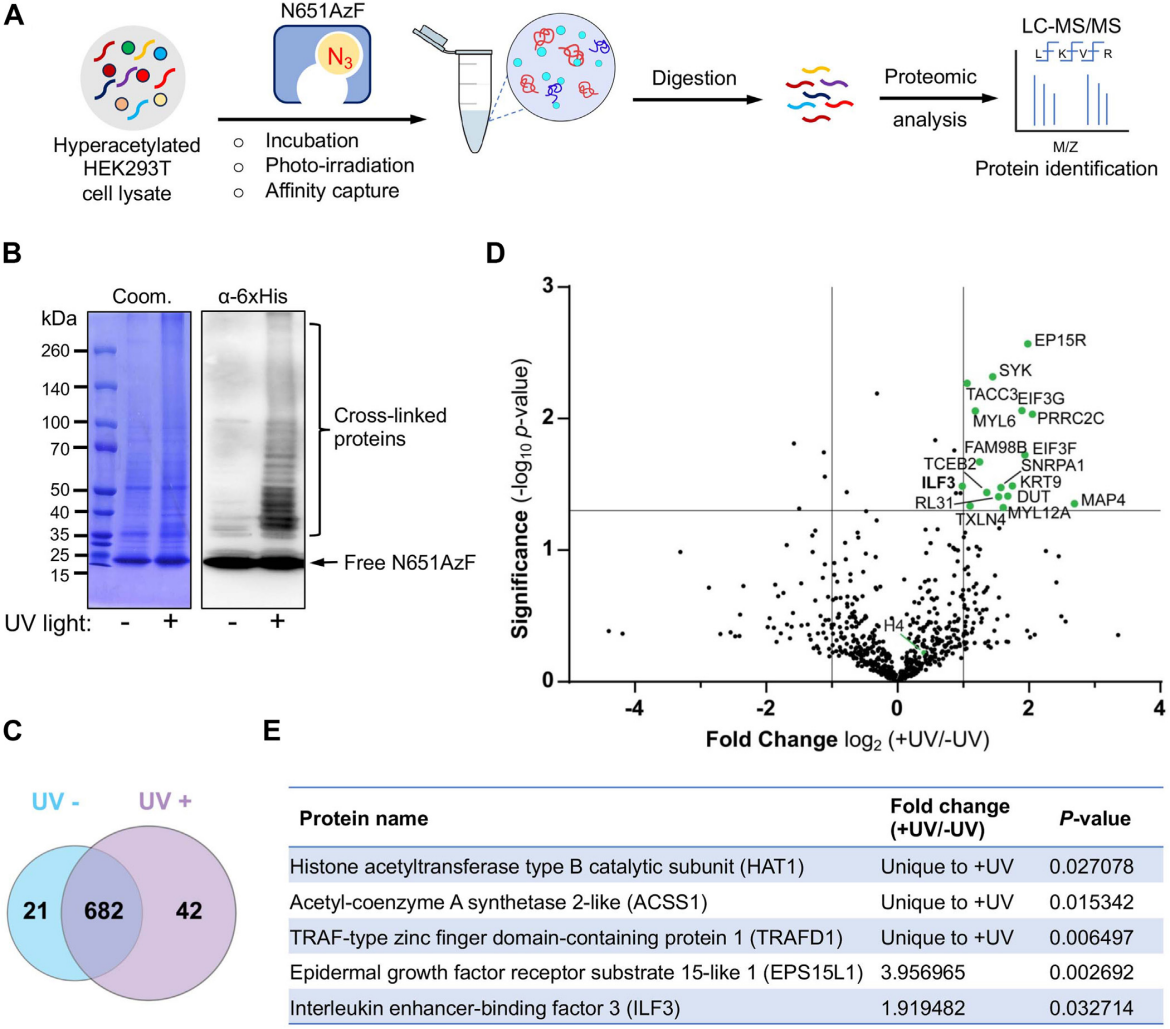
**Proteomic characterization of the BRPF1 interactome.***A*, schematic showing steps involved in cross-linking, enrichment, and characterization of BRPF1 interacting partners present in the cellular proteome. *B*, Coomassie staining and Western blot of the BRPF1 interactome pulled down from HEK293T cell extracts with or without UV light. *C*, *Venn diagram* showing stringently filtered datasets appearing in −UV and +UV treated samples and in both samples. *D*, A *volcano plot* represents potential interacting partners of BRPF1 identified using proteomic analysis. *E*, list of BRPF1 interacting partners identified in the current study. BRPF, bromodomain-PHD finger protein.

Successful cross-linking of N651AzF with acetylated histone H4 peptides and endogenous histone H4 prompted us to profile the non-histone interacting partners of BRPF1 present in human cells. To do so, we generated hyperacetylated proteome by treating the HEK293T cells with deacetylase inhibitor, trichostatin A (Fig. 6*A*). The cell lysate was then incubated with N651AzF and photoirradiated at 365 nm for 15 min. Next, the cross-linked proteins were pulldown using Ni-NTA beads and washed extensively. We observed multiple higher molecular weight protein bands (Fig. 6*B*) on SDS-PAGE gel, indicating successful cross-linking of the engineered BRPF1 with putative non-histone interacting partners. No significant enrichment was observed when the samples were not exposed to UV light.

To characterize the putative interactome of BRPF1, we performed in-solution trypsin digestion of enriched cross-linked proteins (Fig. 6*A*). Subsequently, the digested peptides were subjected to LC-MS/MS. We performed two independent biological replicates of UV-treated and untreated samples to improve confidence in the proteomic analysis. We have employed the label free quantification in MaxQuant tool to analyze the LC-MS/MS data. Mathematica (v10) program was used to further process the “proteinGroups.txt” file output of MaxQuant. The program was used to (1) replace default protein names and symbols with user-defined entries, and removed “potential contaminants” (2) then output file was used to calculate the relative intensity based absolute quantification (riBAQ) values (3), riBAQ values of each protein under a given condition (±UV) were averaged and fold enrichment was calculated, and (4) proteins within a set with a *p*-value of ≤0.05 and fold change ∼2 obtained from a *t* test between control and treated, were considered as "high-confident" bromodomain interactome pool. Applying these filters, we identified 42 proteins present exclusively in the UV-treated samples and 21 in the untreated samples. And 682 proteins were common for both treated and untreated samples, among which 17 were significantly enriched (Fig. 6*C* and Table S5).

Using the IBPP approach, we identified both nuclear and cytosolic proteins as putative interacting partners of BRPF1 (Fig. 6*D* and Table S5). Analysis of the exemplary BRPF1 interactome with high scores (*p*-value of ≤0.05) revealed that the bromodomain interacting partners are associated with a broad spectrum of cellular functions. The interactomes include transcription factors (TCEB2, ILF3); translation-associated factors (EIF3G, EIF3A); RNA-binding proteins such as (SNRPA1), splicing factors (FAM98B, TXLN4), metabolic enzymes (DUT); and scaffolding and structural proteins (MYL6, MAP4). Some important unique to +UV interacting partners are HAT1 enzyme, metabolic enzyme (ACSS1) associated with local acetyl-CoA production, and TRAFD1, a master regulator of INFγ (Fig. 6*E*). These results suggest that the newly identified interactome plays an important role in diverse cellular processes, such as transcription, translation, RNA splicing and stability, cytoskeleton dynamics, and metabolism. Hence, each interactome reported here expands the canonical functions of the BRPF1 protein beyond chromatin and transcription.

### Characterization of acetylated interactome

**Figure 7:**
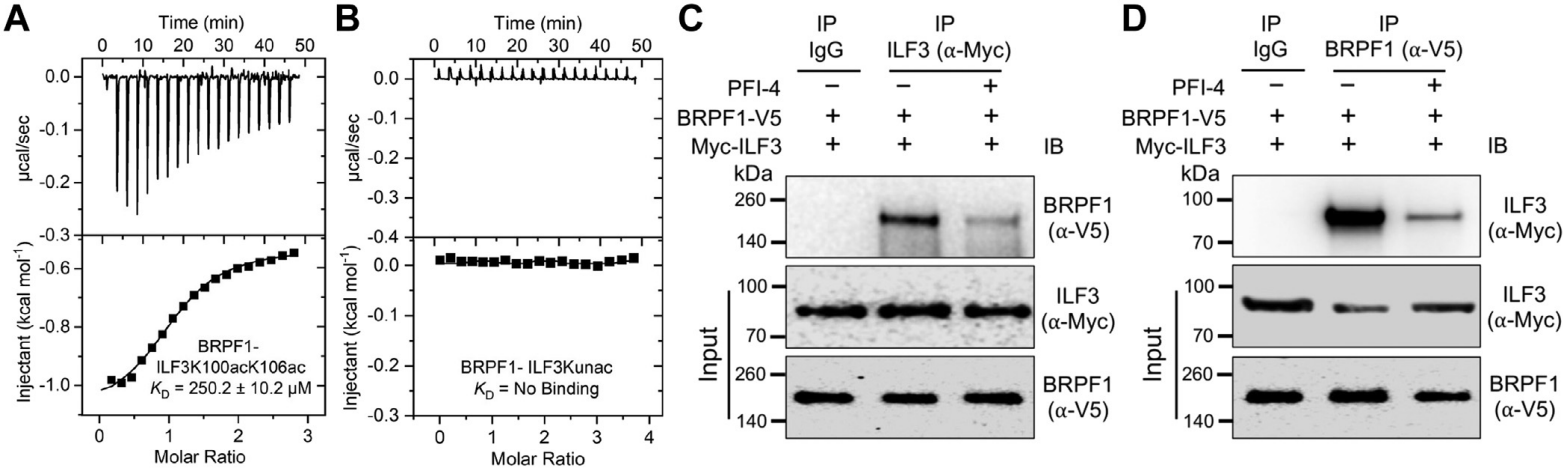
**Interaction of BRPF1 and ILF3.***A* and *B*, ITC measurements for the BRPF1 bromodomain with diacetylated ILF3 peptide 8 and ILF3 unacetylated peptide 9, respectively. *C* and *D*, immunoprecipitation (IP) of BRPF1 and ILF3 in Huh-7 cells. The BRPF1 bromodomain specific inhibitor, PFI-4 significantly reduced the BRPF1–ILF3 interaction. BRPF, bromodomain-PHD finger protei; ILF3, interleukin enhancer–binding factor 3; ITC, isothermal titration calorimetry.

Recent proteomic studies have revealed that a large number of non-histone proteins are acetylated at specific lysine residues and constitute a major portion of the acetylome in mammalian cells (35). However, the biological functions of non-histone protein acetylation remain poorly understood. Our chemoproteomic approach identified some of these acetylated non-histone proteins as interacting partners of the BRPF1 bromodomain. To examine the binding ability of the BRPF1 bromodomain with novel non-histone interacting partners, we selected interleukin enhancer–binding factor 3 (ILF3) as a representative interacting partner identified in this study. ILF3 is reported to be upregulated in several malignancies, including HCC (36, 37). Moreover, analysis of publicly available gene expression data shows that ILF3 is more significantly overexpressed in HCC than the list of BRPF1 interacting partners identified in this study (Fig. S18, *A*–*C*). Interestingly, BRPF1 has also been shown to be frequently upregulated in human HCCs (32) (Fig. S18*B*). Then to validate the BRPF1 and ILF3 interaction, we synthesized a segment of ILF3, RVGLVAKacGLLLKacG 8 (Table S3), known to be acetylated in HEK293T cells, and quantified their affinity (Fig. 7*A* and Table S3) (35). The binding affinity of BRPF1 bromodomain to the ILF3 peptide 8 was determined by ITC binding assay. The WT BRPF1 bromodomain indeed recognized the diacetylated ILF3 peptide 8 with a *K*_D_ value of 250.2 ± 10.2 μM (Fig. 7*A*). No binding was observed with the ILF3 control peptide 9 without acetylation (Fig. 7*B* and Table S3). These results suggests that the BRPF1 bromodomain recognizes diacetylated non-histone ILF3 peptide 8 which is about 10-fold weaker than the diacetylated histone H4 peptide 2 (250.2 μM *versus* 24.6 μM). Notably, the BRPF1-ILF3 binding affinity is within the range in which different bromodomains recognizes their dedicated acetylated histones (38). Then to further validate the BRPF1-ILF3 interaction in the cell, we transiently cotransfected V5-tagged BRPF1 and Myc-tagged ILF3 in Huh-7 HCC cell line and performed immunoprecipitation experiments. We found that Myc-ILF3 immunoprecipitated V5-BRPF1 (Figs. 7*C* and S19*A*) and in reciprocal experiment V5-BRPF1 also immunoprecipitated Myc-ILF3 (Figs. 7*D* and S19*B*). It is noteworthy that addition of PFI-4, a BRPF1 bromodomain specific inhibitor, significantly reduced the BRPF1–ILF3 interaction (Fig. 7C and *D*), recapitulating that the binding of ILF3 with BRPF1 is driven in an acetylation-dependent manner through that BRPF1 bromodomain.

### BRPF1 and ILF3 colocalizes in the genome

**Figure 8:**
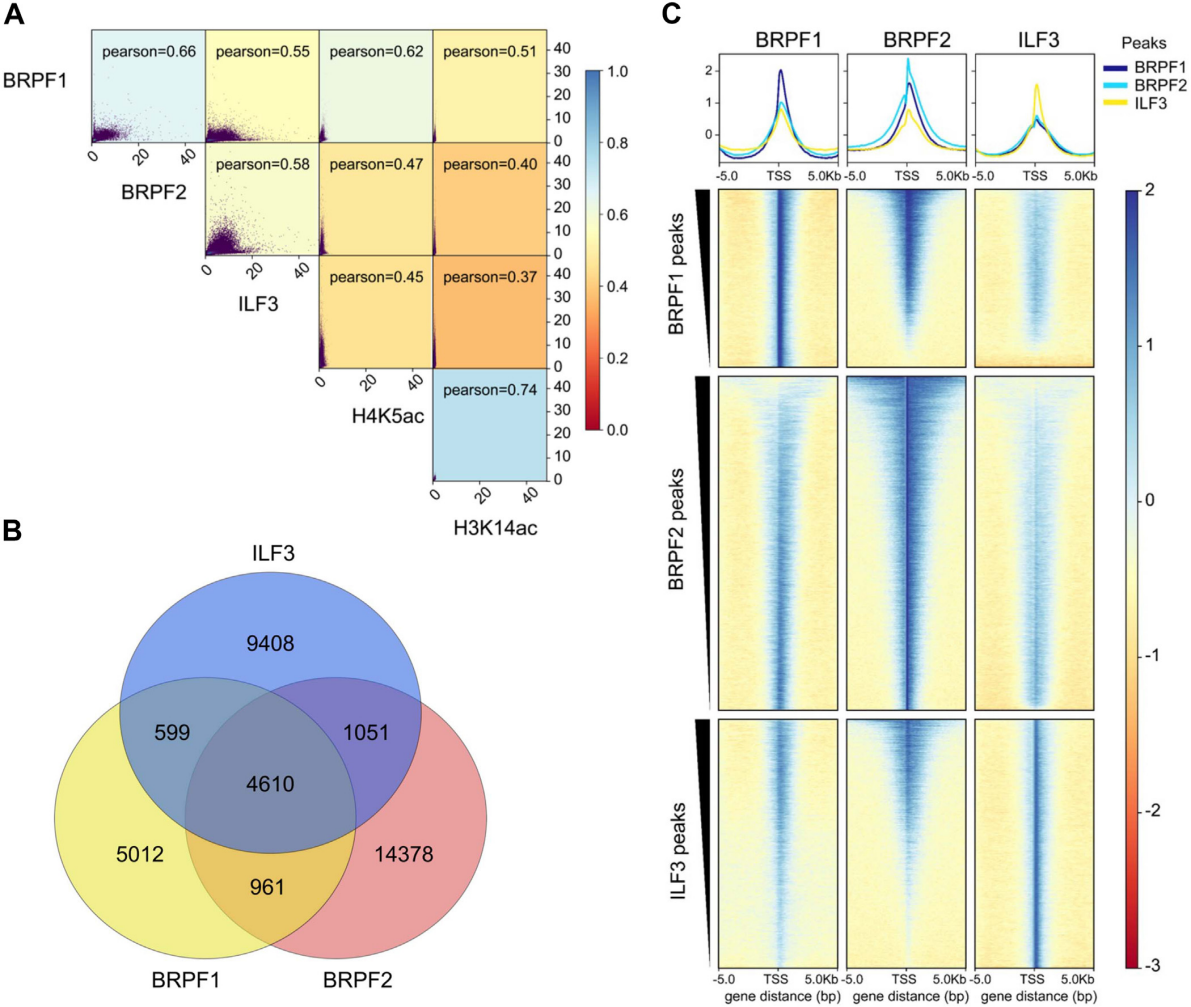
**ChIP-seq analysis.***A*, correlation heat map and scatter plot comparing BRPF1, BRPF2, ILF3, H4K5ac, and H3K14ac localization. *B*, *Venn diagram* showing the overlap of genomic binding sites among BRPF1, BRPF2, and ILF3. *C*, *tornado plot* of BRPF1, BRPF2, and ILF3 ChIP-seq signals to ±5 kb around the TSS of BRPF1, BRPF2, and ILF3-localized genes. Analysis of signals around the gene transcription start sites of BRPF1, BRPF2, and ILF3 localized genes shown in *line graphs* observed in ChIP-seq datasets. Genes were sorted from high to low by peak signal. BRPF, bromodomain-PHD finger protein; ILF3, interleukin enhancer–binding factor 3.

Our *in vitro* experiments demonstrate that the BRPF1 bromodomain transiently binds to the diacetylated ILF3 peptide and also immunoprecipitates ILF3 from cell. To better understand the function of BRPF1 and ILF3 interaction, we looked at their colocalization in the genome. We have also included BRPF2 for the colocalization studies, as it is a paralog of BRPF1. To this end, we looked into the published ChIP-seq datasets of BRPF1 (GSE213695), BRPF2 (GSM1146449), and ILF3 (GSM1418965). The ChIP-seq datasets were processed using a standard pipeline. The trimmomatic tool (0.40) was used to trim the adapter sequences, and the trimmed reads were aligned into the human genome with the Bowtie2 tool (2.5.1), followed by peak calling using MACS2 (2.2.8) for analysis. A comparison of the BRPF1 and BRPF2 peaks with ILF3 and regulatory histone marks H4K5ac and H3K14ac shows that 55% of BRPF1 and 58% of BRPF2 peaks correlate with ILF3 peaks. The correlation of BRPF1 and BRPF2 with ILF3 is comparable with their reported histone ligands H4K5ac and H3K14ac, where the correlation is more than 40% (Fig. 8*A*). Next, the significant peaks of BRPF1, BRPF2, and ILF3 were plotted as a Venn diagram to understand their overlap in the genome. The result demonstrates that approximately 4610 (50%) peaks from BRPF1 overlaps with BRPF2 and ILF3 peaks (Fig. 8*B*). Together these results clearly show that BRPF1 and ILF3, along with BRPF2, colocalizes in the genome.

Tornado plots were created with deepTools to understand the localization of BRPF1, BRPF2, and ILF3 near the transcription start site (TSS) of each of their regulated genes. The analysis showed that ILF3 occupancy is abundant in BRPF1 and BRPF2 localized regions and vice versa (Fig. 8*C*). Next, we performed KEGG pathway analysis to understand the functional significance of BRPF1, BRPF2, and ILF3 colocalization. KEGG enrichment analysis shows that BRPF1, BRPF2, and ILF3 are involved in diverse cell signaling pathways like Rap1, WNT, Hippo, Calcium, MAPK, mTOR, and cAMP, as well as various cancer-associated pathways like basal cell carcinoma, HCC, breast cancer, and gastric cancer (Fig. S20). These results demonstrate that BRPF1, BRPF2, and ILF3 regulate the expression of different genes involved in important cellular processes.

**Figure 9:**
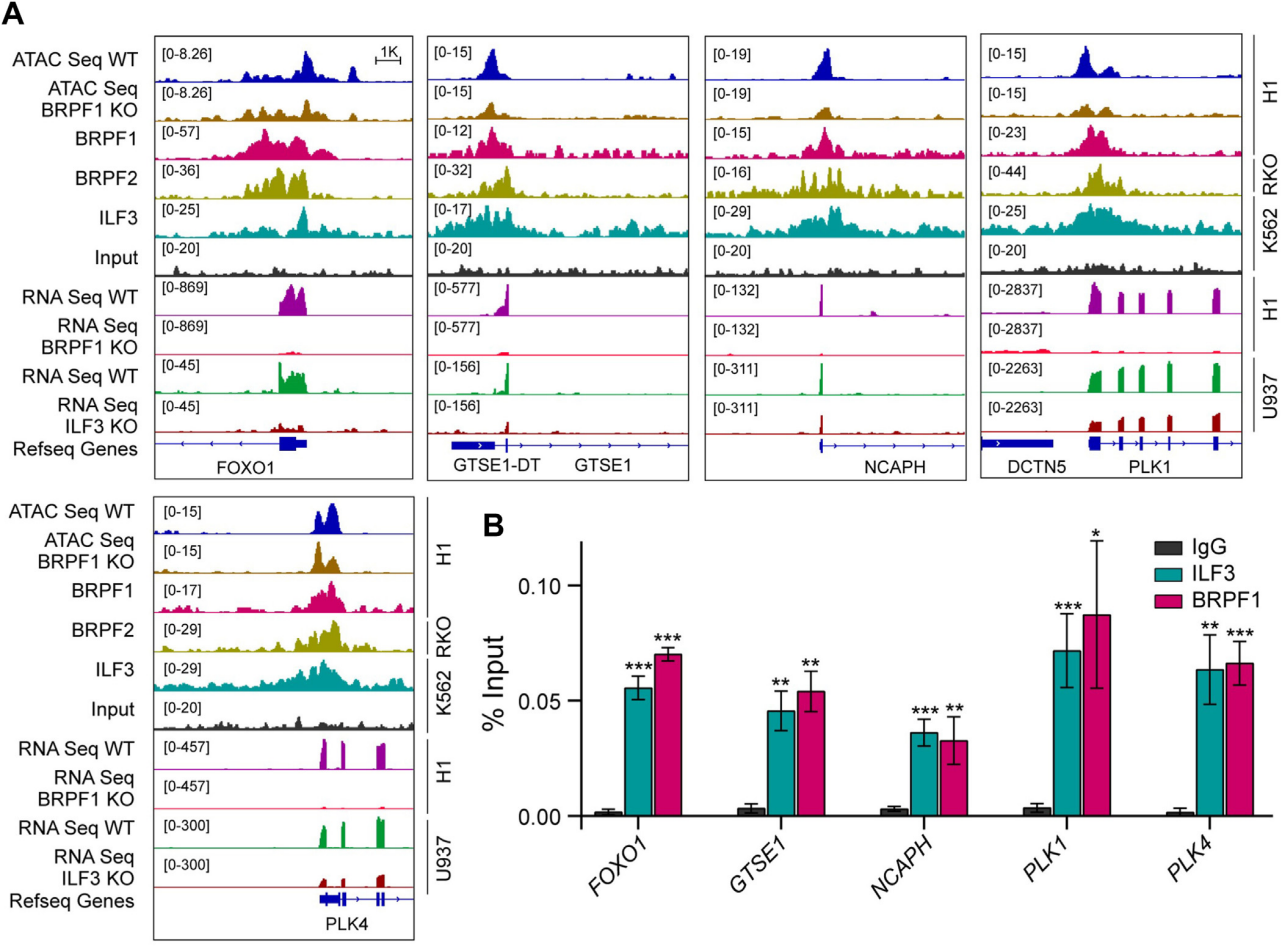
**Visualization of colocalization and expression of HCC hub genes and oncogenic regulators.***A*, IGV snapshots of colocalization of BRPF1, BRPF2, and ILF3 (ChIP-seq) along with chromatin accessibility (ATAC-seq) and gene expression (RNA-seq) of WT and BRPF1 and ILF3 KO conditions at the promoter of HCC hub genes. *B*, ChIP followed by qPCR analysis was performed on the immunoprecipitated DNA using primers designed to amplify regulatory regions of the indicated genes. Data are mean ± SD (n = 3). BRPF, bromodomain-PHD finger protein; HCC, hepatocellular carcinoma; ILF3, interleukin enhancer–binding factor 3.

**Figure 10:**
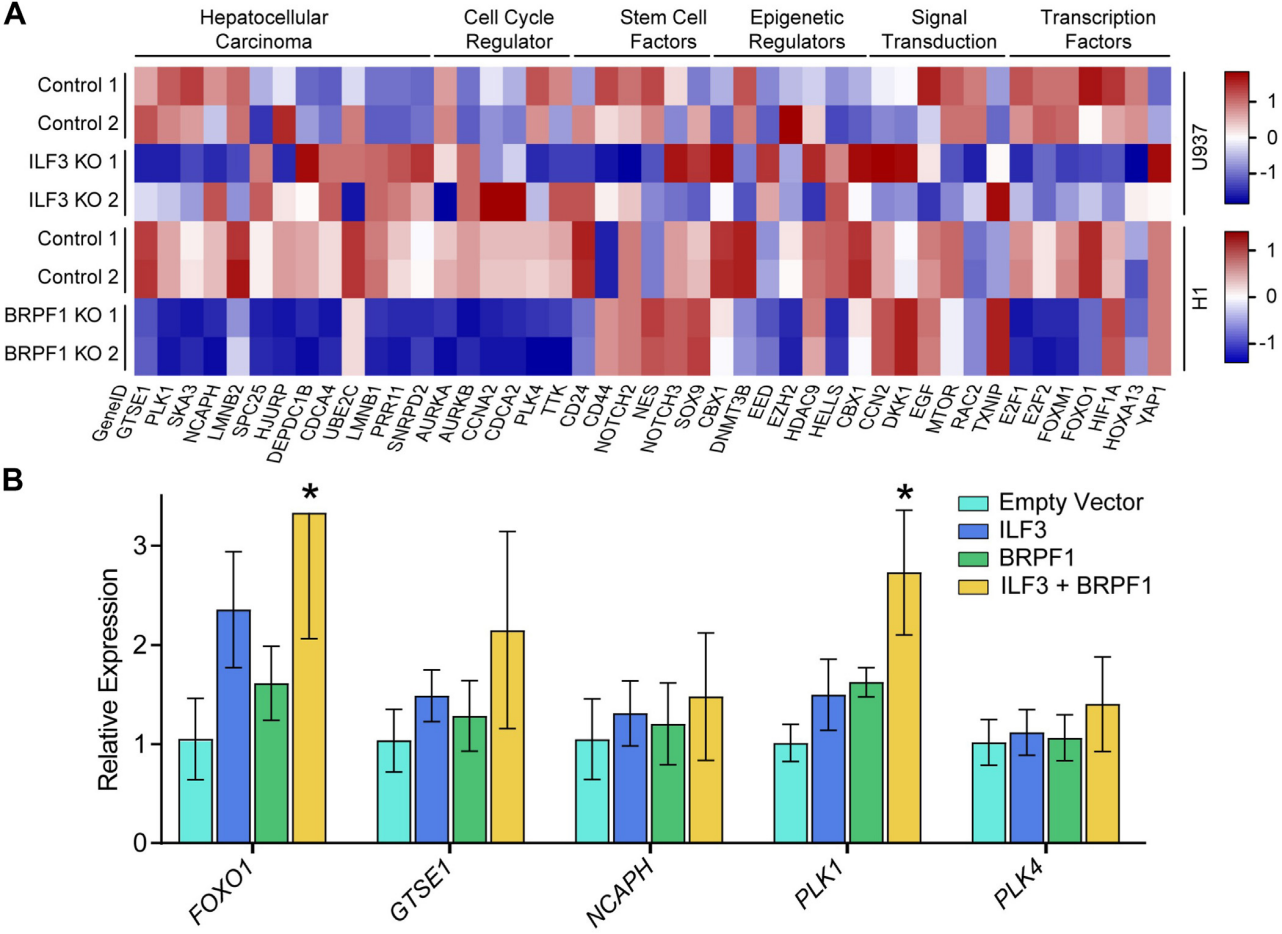
**BRPF1 and ILF3 coregulate expression of crucial genes involved in HCC and cell cycle.***A*, RNA-seq heatmap analysis of the genes associated with HCC and oncogenic regulators in BRPF1 and ILF3 KO conditions. *B*, quantitative RT-PCR (qRT-PCR) results for the differentially expressed transcripts to verify transcriptome profile data analysis. Data are mean ± SD (n = 3). BRPF, bromodomain-PHD finger protein; HCC, hepatocellular carcinoma; ILF3, interleukin enhancer–binding factor 3.

Since previous studies have shown that BRPF1 is overexpressed in HCC (32), we decided to check the colocalization of BRPF1, BRPF2, and ILF3 in the promoter region of HCC-associated hub genes. We observed that BRPF1, BRPF2, and ILF3 localize to TSS of hub genes including GTSE1, PLK1, NCAPH, SKA3, LMNB2, SPC25, HJURP, DEPDC1B, CDCA4, UBE2C, LMNB1, PRR11, and SNRPD2 associated with HCC (Figs. 9, *A* and *B* and S21). To further validate this observation, we performed ChIP-quantitative polymerase chain reaction of five selected HCC hub genes (FOXO1, GTSE1, NCAPH, PLK1, and PLK4) after cotransfecting ILF3 and BRPF1 in Huh-7 cell line and immunoprecipitating the DNA. Our ChIP-qPCR result demonstrates that both BRPF1 and ILF3 significantly localizes in the promoter of these HCC hub genes (Fig. 9*B*). Then, our ATAC-seq analysis shows a reduced chromatin accessibility of HCC hub genes in BRPF1 KO cells (Figs. 9*A* and S21). Next, RNA-seq analysis revealed that some HCC-associated hub genes are downregulated due to KO of BRPF1 and ILF3, but more prominently in the case of BRPF1 KO (Figs. 9*A* and S21). In addition, heatmap analysis of RNA-seq datasets revealed that BRPF1 and ILF3 regulate the expression of HCC hub genes and key oncogenes essential for cancer growth and progression (Fig. 10*A*). Knocking out BRPF1 and ILF3 downregulate expression of HCC associated genes like GTSE1, PLK1, SKA3, LMNB2, cell cycle regulators, AURKA, PLK4, epigenetic factors, DNMT3B, EZH2 and transcription factors, E2F1, E2F2, FOXM1, FOXO1 to name a few (Fig. 10*A*). This observation was further validated by performing qPCR of few selected HCC associated hub genes after transfecting Huh-7 cells with BRPF1 and ILF3. Our qPCR result suggests that in cells transfected with both ILF3 and BRPF1 there is an elevated expression of some genes like FOXO1 and PLK1, while BRPF1 or ILF3 alone could not significantly alter the expression of these genes (Fig. 10*B*). This result implies that BRPF1 and ILF3 are possibly directly interacting to induce expression HCC hub genes and driving tumorigenesis.

### Identification of novel genes regulated by BRPF1 and ILF3

**Figure 11:**
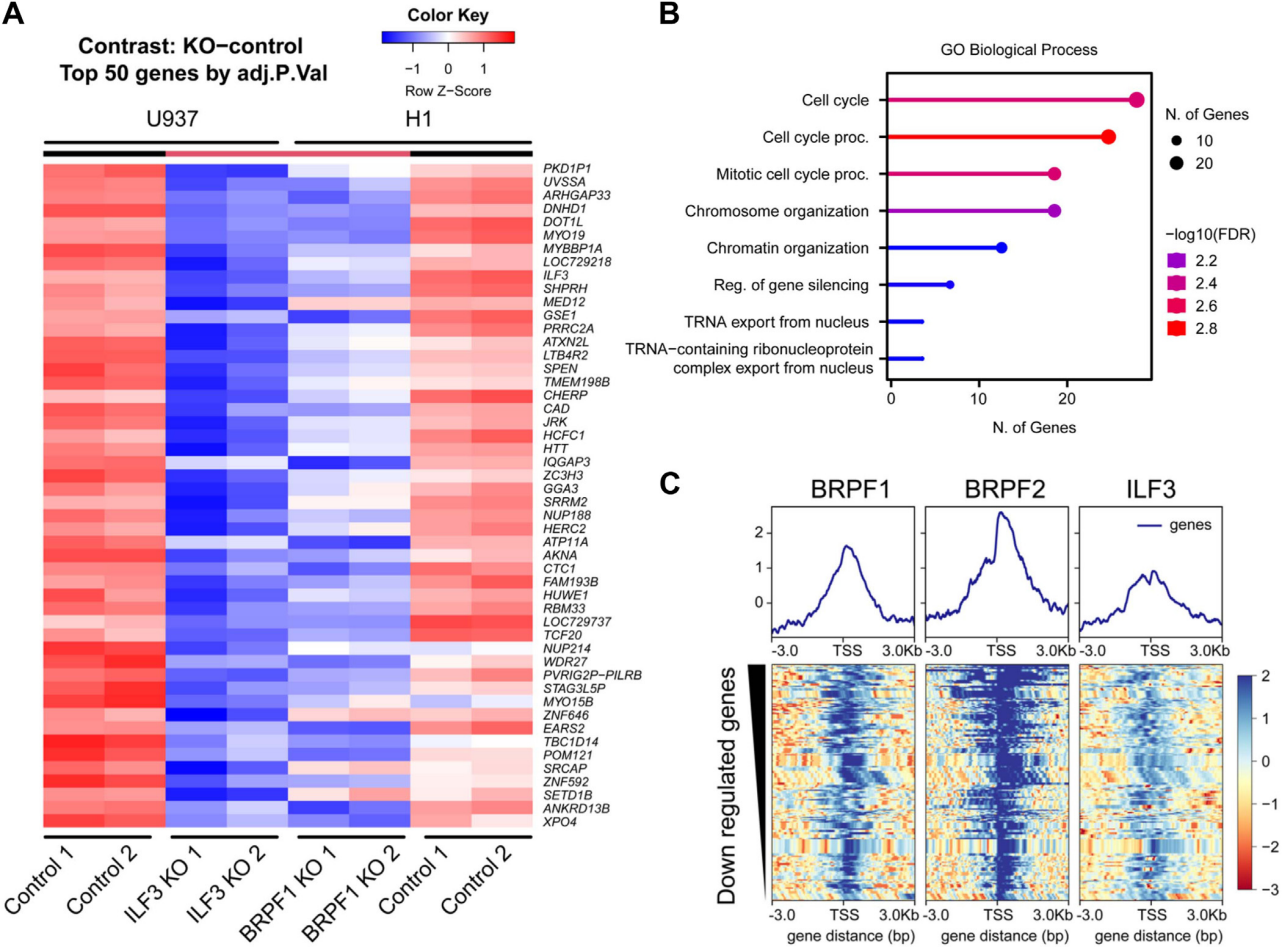
**BRPF1 and ILF3 regulate common genes associated in cellular processes.***A*, heatmap representing RNA-seq analysis of BRPF1 and ILF3 KO condition showing top 50 differentially expressed genes. *B*, GO biological process analysis of the selected top genes demonstrating their function in diverse cellular processes. *C*, *tornado plot* of BRPF1, BRPF2, and ILF3 ChIP-seq signals to ±3 kb around the TSS of the selected top genes from (*A*). BRPF, bromodomain-PHD finger protein; GO, gene ontology; ILF3, interleukin enhancer–binding factor 3.

Our ChIP-seq and RNA-seq data analysis shows that BRPF1 and ILF3 colocalize in the genome and direct the expression of genes associated with HCC and cancer progression (Fig. 10). To better understand the functional relevance of the BRPF1 and ILF3 interaction, we performed a differential gene expression analysis of BRPF1 and ILF3 KO and control cells. We identified top 50 common genes that are regulated by BRPF1 and ILF3, displayed as heat map (Fig. 11*A*). We analyzed the expression of the top 50 genes in independent KO *versus* control cells of BRPF1 and ILF3 and found that these genes are significantly downregulated (Fig. S22). Next, we performed a biological process analysis of the gene ontology of downregulated genes. Our analysis revealed that these genes are mainly involved in cell cycle regulation, chromosomal organization, and regulation of gene expression (Fig. 11*B*). These genes were further analyzed to verify their colocalization with BRPF1 and ILF3 using deepTools (Fig. 11*C*). We found that the downregulated genes are occupied by BRPF1 and ILF3 at their TSS. These data suggest that the BRPF1 and ILF3 interaction is important in regulating genes associated with various cellular processes, including cancer.

## Conclusion

In this work, we site-specifically introduced a photo-cross-linking amino acid AzF carrying an arylazide moiety into the acetyllysine binding pocket of the BRPF1 bromodomain. We selected the best BRPF1 mutant N651AzF based on its binding ability to acetylated histone peptides. In addition, molecular modeling and MD simulation studies revealed detailed insights into the spatial impact of AzF incorporation and acetylated histone peptide binding in the bromodomain. We show that the engineered BRPF1 bromodomain (N651AzF) underwent robust cross-linking with its interacting partners, such as acetylated histone H4 peptides and endogenous acetylated histone H4 upon photoirradiation. Furthermore, N651AzF mutant was employed to capture novel non-histone interacting partners from HEK293T cells. Subsequently, the cross-linked proteins were enriched and characterized by LC-MS/MS. Our proteomics study identified several novel non-histone proteins are the binding partners of BRPF1. More importantly, our literature and proteomic database search results revealed that the majority of the newly identified non-histone proteins are acetylated at lysine residues in the cells (35, 39). The representative example of identified proteins such as ILF3, ACSS1, HAT1, EPS15L1, and TRAFD1 are some of the BRPF1 interactome. Among the above listed proteins, ILF3 is reported to be significantly upregulated in the majority of HCC in comparison with normal tissues (36, 37). Knockdown of ILF3 in HCC cell lines delays cell-cycle progression and inhibits tumor growth *in vivo* (37). Our ITC binding assays demonstrate that the acetylated KGLLLK (100-105) motif in the DZF domain of ILF3 interacts with the BRPF1 bromodomain. And BRPF1 also immunoprecipitated ILF3 from the cell, suggesting that the BRPF1–ILF3 interaction might play an essential role in regulating diverse signaling pathways.

Recent studies demonstrated that BRPF1 is frequently upregulated in human HCCs, and overexpression of BRPF1 is linked to poor survival rates in HCC patients (32). To understand the functional relevance of BRPF1–ILF3 interaction, we analyzed publicly available ChIP-seq datasets. We found that the BRPF1 and ILF3 colocalize in the genome at the TSS of BRPF1-targeted hub genes associated with HCC. Moreover, publicly available RNA-seq data analysis revealed that a few critical HCC-associated genes are downregulated under the KO conditions of BRPF1 and ILF3. Our study provides a potential mechanistic link between BRPF1 and ILF3 interaction and their role in developing HCCs. This data will be crucial to design potent bromodomain-specific small-molecule inhibitors of BRPF1, a therapeutic target for HCC treatment. We anticipate that future experiments with the non-histone interacting partners identified in this study will be immensely helpful to gain a detailed understanding of BRPF1 in transcriptional regulation, brain development, adult medulloblastoma, and leukemogenesis.

## Experimental procedures

### General materials, methods, and equipment

All of the plasmids for bacterial expression were obtained as gifts from individual laboratories or purchased from addgene (Table S1). Mutagenic primers were obtained from Sigma-Aldrich (Table S2). Commercially available competent bacterial cells were used for protein expression and mutagenesis. HEK293T cells were obtained from American Type Culture Collection and used following the manufacturer’s protocol. All of the antibodies used in this study were purchased from established vendors and used following the manufacturer’s protocol. AzF 1 was a kind gift from K. Islam (University of Pittsburgh) synthesized and characterized as reported previously (26). All histone and non-histone peptides 2 to 9 were synthesized by GL Biochem Ltd and S. Biochem Ltd and purified by HPLC to 98% purity (Figs. S2–S17 and Table S3). The concentrations of unlabeled peptides 2, 3, 8, and 9 were determined on the basis of the observation that 1 mg/ml peptide generates an absorbance (*A*_205_) of 30 at 205 nm (40). The concentrations of TAMRA-labeled peptides 4 to 7 were determined by measuring the absorbance at a wavelength of 555 nm with an extinction coefficient of 65,000 L^−1^ cm^−1^ M^−1^. The integrity of the purified peptides was confirmed by MALDI-MS.

### Mutagenesis, expression, and purification of the BRPF1 bromodomain

Protein expression and purification were performed using previously described methods (30, 41). The N-terminal 6xHis-tagged BRPF1 bromodomain plasmid (a gift from Nicola-Burgess-Brown, addgene catalog no. 53620) was transformed into One Shot BL21 star (DE3) *Escherichia coli* competent cells (Invitrogen, catalog no. C601003) using pNIC28-Bsa4 kanamycin-resistant vectors. A single colony was picked up and grown overnight at 37 °C in 10 ml of LB broth in the presence of 50 μg mL^−1^ kanamycin. The culture was diluted 100-fold and allowed to grow at 37 °C to an absorbance (*A*_600_) of 1. Protein expression was induced overnight at 17 °C with 0.5 mM IPTG in an Innova 44 Incubator shaker (New Brunswick Scientific). Proteins were purified as follows. Harvested cells were resuspended in 15 ml of lysis buffer (50 mM Hepes [pH 7.5], 300 mM NaCl, 5 mM β-mercaptoethanol, 5% glycerol, 25 mM imidazole, lysozyme, DNase, and 1:200 (v/v) Protease Inhibitor Cocktail III [Calbiochem]). The cells were lysed by pulse sonication and centrifuged at 13,000 rpm for 40 min at 4 °C. According to the manufacturer’s instructions, the soluble extracts were subjected to Ni-NTA agarose resin (QIAGEN, catalog no. 30210). After 20 volumes of wash buffer (50 mM Hepes [pH 7.5], 300 mM NaCl, 5 mM β-mercaptoethanol, 5% glycerol, and 25 mM imidazole) had been passed through the column, proteins were eluted with a buffer containing 50 mM Hepes (pH 7.5), 300 mM NaCl, 5 mM β-mercaptoethanol, 5% glycerol, and 250 mM imidazole. Proteins were further purified by gel-filtration chromatography (Superdex-75) using an AKTA pure FPLC system (GE Healthcare) with a buffer containing 50 mM Hepes (pH 7.5), 200 mM NaCl, and 5% glycerol. Purified proteins were concentrated using an Amicon Ultra-10 k centrifugal filter device (Merck Millipore), and the concentration was determined using a Bradford assay kit (Bio-Rad Laboratories) with bovine serum albumin as a standard. The proteins were aliquoted and stored at −80 °C until use.

Site-directed mutagenesis was performed to generate the BRPF1 bromodomain amber codon (TAG) mutants N651TAG and V662TAG using the QuikChange Lightning Site-Directed Mutagenesis Kit, and the resulting mutant plasmids were confirmed by DNA sequencing. To express BRPF1 bromodomain variants carrying AzF 1 at site-specific positions, BRPF1 amber variants (N651TAG and V662TAG) and pEVOL-pAzF (a kind gift from K. Islam, University of Pittsburgh, addgene catalog no. 31186) were cotransformed into One Shot BL21 Star (DE3) *E. coli* competent cells. After transformation, cells were recovered in 200 μl of SOC medium and incubated in a 37 °C shaker for 1 h at 225 rpm before being plated on a dual antibiotic LB Miller agar plate containing 50 μg mL^−1^ kanamycin and 35 μg mL^−1^ chloramphenicol. A single colony was picked up and inoculated into 5 ml of LB Miller broth in the presence of 50 μg mL^−1^ kanamycin and 35 μg mL^−1^ chloramphenicol and grown overnight in a 37 °C shaker at 225 rpm. This overnight culture was centrifuged (Eppendorf Centrifuge 5910 R, S-4xUniversal swing-bucket rotor) at 1000*g* (2158 rpm) for 10 min at room temperature (RT), and the supernatant (LB Miller broth) was discarded. The collected cell pellet was then resuspended in 1 ml of M9 medium and used to inoculate 500 ml of GMML medium supplemented with 50 μg mL^−1^ kanamycin and 35 μg mL^−1^ chloramphenicol. Cells were allowed to grow at 37 °C in an incubator shaker (225 rpm) to an absorbance (*A*_600_) of 1. The unnatural amino acid (AzF 1) was prepared by dilution in 5 ml of sterilized deionized water, and that mixture was added to the bacterial culture to a final concentration of 1 mM. Then the cells were cooled to 17 °C and allowed to grow for 30 min; at this stage, AzF-specific aminoacyl-tRNA synthetase expression was induced with 0.05% (w/v) arabinose and allowed to shake for an additional 30 min at 17 °C. Finally, BRPF1 protein expression was induced by the addition of 0.5 mM IPTG and cells were allowed to grow for 20 h at 17 °C. The BRPF1 bromodomain mutants were purified as described above while minimizing the exposure to ambient light. The purified proteins were subjected to MALDI-MS to confirm the incorporation of AzF 1.

### Isothermal titration calorimetry

ITC binding experiments were performed as previously described (30, 41). Briefly, experiments were carried out on a MicroCal PEAQ ITC instrument (Malvern) at 15 °C while the sample was being stirred at 750 rpm. Buffers of protein and peptides were matched to 50 mM Hepes (pH 7.5), 200 mM NaCl, and 5% glycerol. Each titration comprised one initial injection of 0.4 μl lasting 0.8 s, followed by 18 injections of 2 μl lasting 4 s each at 2.5 min intervals. The initial injection was discarded during data analysis. The microsyringe (40 μl) was loaded with a peptide sample solution at a concentration of 1.5 to 1.8 mM. It was injected into the cell (200 μl), occupied by a protein concentration of 80 to 100 μM. All of the data were fitted to a single-binding site model using the MicroCal PEAQ ITC analysis software to calculate the stoichiometry (*N*), binding constant (*K*_D_), enthalpy (*ΔH*), and entropy (*ΔS*) of the interaction. The final titration figures were prepared using OriginPro 2020 (OriginLab).

### Molecular modeling and MD

The computational modeling and MD simulation were performed as previously described with slight modifications (27, 30, 42). Briefly, the crystal structure of BRPF1 bromodomain (PDB entry 4LC2) was obtained from the RCSB Protein Data Bank (RCSB PDB). Next, we prepared the BRPF1–H4K5acK8ac peptide complex by superimposing it with the BRD4 BD1–H4K5acK8ac complex (PDB entry 3UVW) using PyMOL software package. The targeted amino acids of the BRPF1 were replaced with AzF using the PyMOL builder and the MMFF94 structural optimization algorithm for initial structure approximation. All AzF mutants were then subjected to MD simulations with GROMACS (https://www.gromacs.org) (2018.3) (43) using CHARMM27 all-atom force field (44) modified according to the parameters developed by Smith *et al* (45). Then the AzF-modeled mutant proteins were solvated in a dodecahedral box with a distance of 1 nm between the protein and edge of the solvated box. The solvated system was neutralized by adding sodium during the simulation. To ensure the complex's steric clashes or geometry, the system was energy minimized using the LINCS constraints and steepest descent algorithms (5000 steps and a force of <1000 kJ mol^−1^ nm^−1^), followed by system equilibration under *NVT* and *NPT* ensembles. After the energy minimization and equilibration phase, the system was subjected to production MD for 100 ns by following the same time steps described above. To analyze the effect of AzF incorporation, the RMSD and rms fluctuation was calculated for each α-carbon of the protein. All plotting was done in xmgrace and GraphPad Prism. MD snapshots are extracted and analyzed using PyMOL.

### Photo-cross-linking experiment with histone peptides and in-gel fluorescence

Photo-cross-linking of BRPF1 bromodomain mutants with acetylated histone peptides was performed by following the method previously described (26, 27, 30). Briefly, 5 μM histone H4 peptides 4 to 7 carrying a TAMRA at the C terminus were preincubated with 50 μM BRPF1 bromodomain mutants (N651AzF and V662AzF) in 100 μl of binding buffer (50 mM Hepes [pH 7.5], 150 mM NaCl, and 0.001% Tween 20) for 30 min at 4 °C on a rotator. After incubation, the samples were split into two 50 μl volumes in clear PCR tubes (Axygen). The sample-containing PCR tubes were placed into semimicro visible cuvettes (300–900 nm, Eppendorf, catalog no. 0030079353) filled with ice cubes to maintain the temperature and irradiated with UV light (365 nm, 8 W lamps, model ECX-F20.L V1, Vilber Lourmat) for 3 × 10 min at 4 °C. Negative samples were not subjected to UV irradiation and stored in the dark at 4 °C. After irradiation, the samples were placed in 1.5 ml tubes having 50 to 60 μl of Ni-NTA agarose beads (QIAGEN) pre-equilibrated with the buffer (50 mM Hepes [pH 7.5], 150 mM NaCl, and 0.001% Tween 20), and the samples were incubated for 1 h at 4 °C on a rotator. The un-cross-linked histone peptides were removed by washing the samples with washing buffer (50 mM Hepes [pH 7.5], 400 mM KCl, and 0.5% Triton X-100) five times at RT. The proteins were eluted with 30 μl of elution buffer containing 50 mM Hepes (pH 7.5) and 500 mM imidazole and incubated for 5 min at RT with intermittent mixing. The eluted proteins were mixed with 10 μl of 1× TruPAGE LDS sample buffer (Sigma, catalog no. PCG3009) and heat-denatured at 95 °C for 10 min. Finally, the cross-linked proteins were separated on a 12% SDS-PAGE gel and imaged on a G:BOX Chemi XRQ gel doc system (Syngene) using the TAMRA fluorophore excitation wavelength. The gel was subsequently stained with a Coomassie brilliant blue R-250 staining solution to confirm the presence of proteins in all of the samples.

### Mammalian cell culture

HEK293T cells were grown in Dulbecco's modified Eagle's medium supplemented with 10% FBS and antibiotics (penicillin/streptomycin cocktail) in a humidified atmosphere containing 5% CO2. Cells at ∼90% confluence were treated with 2 μM histone deacetylase inhibitor, trichostatin A (Sigma, catalog no. T8552) dissolved in dimethyl sulfoxide to generate hyperacetylated histones. Twenty hours posttreatment, medium was removed and the cells were rinsed with cold PBS. The harvested cells were rewashed with cold PBS and frozen as a dry pellet at −80 °C.

### Acid extraction of histones

Histones were extracted from HeLa cells using the acid-extraction protocol as previously described (46). Briefly, frozen cell pellets from T75 flask of HeLa cells were resuspended in 1 ml of hypotonic lysis buffer (10 mM Tris–HCl pH 8.0, 1 mM KCl, 1.5 mM MgCl_2_, 1 mM DTT, 1 mM PMSF, and protease inhibitor cocktail) and incubated for 30 min on rotator at 4 °C. Samples were then centrifuged (10,000*g*, 10 min at 4 °C), and the supernatant was discarded entirely. The nuclei resuspended in 800 μl of 0.4 N H_2_SO_4_, vortexed intermittently for 5 min, and further incubated at 4 °C on a nutator for overnight. The nuclear debris was pelleted by centrifugation (16000*g*, 10 min at 4 °C), and the supernatant containing histones were collected. The histones were precipitated by adding 264 μl trichloroacetic acid drop by drop to histone solution and invert the tube several times to mix the solutions and incubated the samples on ice for 30 min. Finally, histones were pelleted by centrifugation (16000*g*, 10 min at 4 °C), and the supernatant was discarded. The histones pellet was washed twice with ice-cold acetone, followed by centrifugation (16000*g*, 5 min at 4 °C), and carefully removed the supernatant. The histone pellet was air-dried for 20 min at RT and subsequently dissolved in an appropriate volume of ddH2O and transferred into a fresh tube. The aliquoted histones were stored at −80 °C before use.

### Photo-cross-linking with hyperacetylated histone H4 and Western blotting

Photo-cross-linking with histone extracts was performed by methods as previously described with slight modifications (26, 27). Briefly, 20 to 25 μg hyperacetylated histones (extracted from HEK293T cells) were incubated with 50 μM BRPF1-N651AzF mutant in 50 μl binding buffer (50 mM Hepes pH 7.5, 150 mM NaCl, 0.001% Tween 20, and 1 mM tris (2-carboxyethyl)phosphine for 30 min at 4 °C on a rotator. After incubation, the samples were cross-linked as described above. Finally, the cross-linked proteins were separated on a 12%-SDS-PAGE gel and transferred onto a 0.45 μm PVDF membrane at a constant voltage of 80 V for 1.5 h at 4 °C. The membrane was rinsed in TBST buffer (50 mM Tris pH 7.4, 150 mM NaCl, and 0.1% Tween-20) and blocked for an hour at RT in 5% milk buffer prepared in TBST. Immunoblotting was performed with the following primary antibodies: anti-H4K5ac (Invitrogen, catalog no. MA532009) and anti-6xHis (Invitrogen, catalog no. MA121315) overnight at 4 °C. The membranes were washed with TBST buffer thrice at RT for 5 min each. The blots were then incubated with the horseradish peroxidase-conjugated secondary antibodies Goat anti-Rabbit IgG (Invitrogen, catalog no. 31466) or Goat anti-mouse IgG (Invitrogen, catalog no. 31431) with 5% nonfat dry milk, dilution 1:5000 in TBST. The membranes were rewashed with TBST buffer thrice at RT for 5 min each. Protein bands were visualized by chemiluminescence using SuperSignal West Pico PLUS substrate (Invitrogen, catalog no. 34577) following the manufacturer’s protocol.

### Preparation of HEK293T cell lysate

Twenty hours posttreatment with deacetylase inhibitor (trichostatin A), media was removed and rinsed the cells with cold PBS buffer. The harvested cells were resuspended in ice cold lysis buffer containing 25 mM Tris–HCl pH 7.4, 150 mM NaCl, 1% NP-40, 1 mM EDTA, 5% glycerol (Pierce IP lysis buffer, catalog no. 87787), and supplemented with 1× Pierce protease inhibitor cocktail and incubated on ice for 5 min with periodic mixing. Cell lysate was centrifuged at 13,000*g* for 10 min at 4 °C to pellet the cell debris. The supernatant was transferred into fresh microcentrifuge tube, and the protein concentration was determined by Bradford assay (Bio-Rad Laboratories). The supernatant containing cell lysate was used for subsequent photo-cross-linking and Western blotting experiments.

### Photo-cross-linking with HEK293T cell lysate and Western blotting

For photo-cross-linking studies, ∼1 mg of HEK293T cell lysate was incubated with 50 μM BRPF1-N651AzF mutant in a buffer containing 50 mM Hepes pH 7.5, 150 mM NaCl, 0.001% Tween 20, and 1 mM tris (2-carboxyethyl)phosphine. After 1 h of incubation at RT, the samples were subjected to UV irradiation at 365 nm for 30 min at 4 °C. Negative controls were not subjected to UV exposure. Samples were then bound to Ni-NTA agarose resin and incubated for 1 h at 4 °C with gentle rotation. To remove un-cross-linked proteins, present in cell lysates, samples were washed five times with washing buffer (50 mM Hepes pH 7.5, 400 mM KCl, 5% Triton X-100). The cross-linked proteins were eluted in 30 μl elution buffer containing 50 mM Hepes pH 7.5, 500 mM imidazole, and incubated for 5 min at RT with intermittent mixing. The eluted proteins were separated on a 4 to 15% Mini-PROTEAN TGX precast SDS-PAGE gel (Bio-Rad, #4561085) and transferred onto a 0.45 μm PVDF membrane at a constant voltage of 80 V for 1.5 h at 4 °C. The membrane was rinsed in TBST buffer (50 mM Tris pH 7.4, 150 mM NaCl, and 0.1% Tween-20) and blocked for an hour at RT in 5% milk buffer prepared in TBST. Immunoblotting was anti-6xHis tag antibody (Invitrogen, catalog no. MA121315) overnight at 4 °C. The membrane was washed with TBST buffer thrice at RT for 5 min each. The blots were then incubated with the horseradish peroxidase-conjugated secondary antibody Goat anti-mouse IgG (Invitrogen, catalog no. 31431) with 5% nonfat dry milk, dilution 1:5000 in TBST. The membrane was rewashed with TBST buffer thrice at RT for 5 min each. Protein bands were visualized by chemiluminescence using SuperSignal West Pico PLUS substrate (Invitrogen, catalog no. 34577) following the manufacturer’s protocol.

### LC-MS/MS analysis

In-solution trypsin digestion was carried out by following the standard protocol. Proteolytic peptides from in-solution trypsin digestion were analyzed by a nanoflow reverse-phased LC-MS/MS. Tryptic peptides were loaded onto a PepMap RSLC C18 column (2 μm, 100 Å × 50 cm) of the LC system (Q-Exactive Plus Biopharma, Thermo Fisher Scientific). Chromatographic separation was performed using a binary solvent system (solvent A: water with 0.1% formic acid; solvent B: acetonitrile and water in 85:15 ratios with 0.1% formic acid). MaxQuant v2.0.3.0 software (https://www.maxquant.org) was used for protein identification and quantitation. Mass spectrometry data were searched against UniProt-proteome-UP000005640 using Andromeda. Protein identifications were reported with a false discovery rate of 1%. We used MaxQuant to calculate iBAQ, a measure of protein abundance.

#### Additional mass spectrometry data processing

The mass spectrometry data processing pipeline were performed as previously described by Barr-Gillespie with slight modifications (47). Mathematica v10 program was used to further process the MaxQuant “proteinGroups.txt” file. The program was used to (1) replaced default protein names and symbols with user-defined entries (2), delete all the entries that are marked by MaxQuant as a “potential contaminant” or “Reverse” (proteins labeled as “only identified by site” were retained) (3), group together all proteins that share >30% of their peptides, and (4) prepare an output file. The output file was imported into Excel format, where we calculated the riBAQ, which is the iBAQ (present in both replicates) divided by the total iBAQ value of the respective groups.

### Huh-7 cell culture and transfection

Huh-7, human HCC cells (were a kind gift from Dr Arnab Gupta, IISER Kolkata) were grown in Dulbecco's modified Eagle's medium with 10% FBS, and 1× antibiotic-antimycotic in a humidified atmosphere containing 5% CO_2_. Cells were transfected at 80 to 90% confluency with a ratio of 1 μg DNA per 1 × 10^6^ cells for pcDNA6.2-BRPF1-V5 (addgene, catalog no. 65382), and pcDNA3-Myc-ILF3 (was a kind gift from Philippe P. Roux, University de Montreal). BRPF1 and ILF3. A 2:1 ratio of Lipofectamine 2000 (Invitrogen, catalog no. 11668019) to DNA was used. First DNA and lipofectamine were incubated separately for 5 min at RT in Opti-MEM (Gibco, catalog no. 31985070). Then the DNA and lipofectamine were mixed together and incubated at RT for 20 min and then added to the cells. Six hours posttransfection, media was replaced and treated with deacetylase inhibitor trichostatin A (Sigma, catalog no. T8552) to a final concentration of 2 μM. Cells were collected after 24 h posttransfection and frozen as a dry pellet at −80 °C before use.

### Immunoprecipitation and Western blotting assays

The BRPF1 and ILF3 overexpressed Huh-7 cells were collected, washed twice with PBS and centrifuged at 300*g* for 5 min at RT. Cell pellets are resuspended in IP lysis buffer (Invitrogen, catalog no. 87787) supplemented with 1× protease inhibitor cocktail and incubated at 4 °C with a rotation for 5 min. Cell debris was removed by centrifugation at 13000*g* for 10 min at 4 °C. Immunoprecipitation was performed by using Dynabeads Protein G Immunoprecipitation Kit (Invitrogen, catalog no. 10007D) according to the manufacturer protocol. Dynabeads Protein G magnetic beads were incubated with 2.5 μg anti-V5 antibody (CST, catalog no. 13202S), 2.5 μg anti-Myc antibody (CST, catalog no. 2276S), or control IgG antibody (CST, catalog no. 5415) for 30 min at RT with rotation in Ab binding and washing buffer in presence and absence of 5 μM PFI-4 inhibitor (Sigma, catalog no. PZ0307). After pelleting and washing beads, cell lysates were added to Ab-conjugated magnetic beads and incubated overnight at 4 °C with rotation. The beads were washed three times with the washing buffer and transferred to a new microcentrifuge tube. Samples were eluted with 20 μl elution buffer and 10 μl TruPAGE LDS sample buffer and heat denatured at 70 °C for 10 min. The immunoprecipitated proteins were separated on 8% SDS-PAGE and analyzed by Western blotting.

### Public ChIP-seq, RNA-seq, and ATAC-seq datasets

ChIP-seq data for BRPF1 in H1 cells were obtained from GEO; accession number GSE213695. ChIP-seq datasets of BRPF2 in RKO (GSM1146449) and ILF3 in K652 (GSM3634237), including inputs, GSM818283 of RKO and GSM2423771 of K562 cell line were also gathered from GEO project. ATAC-seq datasets of H1 cell were obtained from GSE213690. RNA-seq datasets for BRPF1 was downloaded from GSE213694 and ILF3 was downloaded from GSE154257.

### Processing and analysis of ChIP-seq data

Raw ChIP-seq fastq file was downloaded, processed, and analyzed by using public Galaxy server environment and in house tools (48). At first, for verifying the sequencing quality and adapter trimming, FastQC and Trimmomatic tool was used (49). Trimmed fastq reads were then aligned and mapped in paired-end mode by utilizing the bowtie2 (v2.4.2) tool (50), and *Homo sapiens* (human) genome assembly GRCh38 was used as the reference genome. The aligned reads were subsequently used for peak calling using the MACS2 (v2.1.1) tool over input (51). Then irreproducibility discovery rate analysis process was adopted to get the final sets of peaks. Irreproducibility discovery rate threshold of 0.05 was selected to acquire the final set of standard peaks. The ENCODE guidelines were adapted to discard the peaks overlapping with the blacklisted regions. We used integrative genomics viewer to visualize the genomic context of the collected and processed ChIP-seq datasets. Subsequent analysis was accomplished using R (v3.4.0)/Bioconductor environment, and the plots were generated based on the ChIPseeker library (52). The deepTools (v3.5.2) was used to generate tornado and line plots (53).

### Processing and analysis of RNA-seq data

We analyzed the RNA-seq data by following the Galaxy tutorials. The raw reads were processed, and filtered the adapter sequences by the Cutadapt tool (v3.4). Trimmed reads were aligned to the human reference genome (hg38) using Hisat2 (v2.0.4) with default parameters. SAMtools (v1.3.1) and featurecount (v0.6.0) were used to calculate gene expression profiles. The raw counts were filtered (a threshold of at least ten average raw read counts among samples) and normalized using Limma-voom (Galaxy v3.50.1 + galaxy0) tools. Then, the differential expression and principal component analysis analysis were performed. Differentially expressed genes were considered significant if their *p*-value <0.01 and a fold-change >1.5. Next, the heatmap is generated using the selected gene sets in the galaxy server using the heatmap2 tool. Functional analysis of the gene sets was done by ShinyGo online tool.

### Processing and analysis of ATAC-seq data

Adapter sequences were removed from the raw data using cutadapt (v3.4) and aligned to the human genome (UCSC hg38) using Bowtie2 (v2.4.2). Duplicate reads were removed using SAMtools (v1.3.1) and Picard tools (v2.2.4). Signals were compiled using MACS2 (v2.1.1) call peak and bdgcmp, and differential open peaks were called using MACS2 bigdiff. Open chromatin localization was visualized using integrative genomics viewer tools.

### ChIP-qPCR

ChIP experiments were performed as previously reported with slight modifications (54). Briefly, the Huh-7 cells were transfected with BRPF1 and ILF3 were collected and fixed in 1% formaldehyde for 20 min, and quenched with 125 mM Glycine for 5 min at RT. The cells were thoroughly washed with PBS supplemented with protease inhibitors. The pellets were resuspended in a high salt lysis/sonication buffer (25 mM Tris–HCl pH 7.5, 0.5% sodium deoxycholate, 0.1% SDS, 1% Triton X-100, 5 mM EDTA, 800 mM NaCl and protease inhibitors) and sonicated using Qsonica Q700 (pulsed 5 s on, 60 s off, for 30 s processing time). Insoluble cell debris was pelleted *via* centrifugation at 13,600*g* for 30 min at 4 °C. Dynabeads Protein G were incubated with 2.5 μg anti-V5 antibody (CST, catalog no. 13202S), 2.5 μg anti-Myc antibody (CST, catalog no. 2276S), or control IgG antibody (CST, catalog no. 5415) for 30 min at RT with rotation in Ab binding and washing buffer. The supernatant containing the soluble chromatin was incubated with Ab-conjugated magnetic beads at 4 °C for overnight, with rotation. Next, the beads were washed with Wash Buffer A (50 mM Hepes pH 7.9, 140 mM NaCL, 1 mM EDTA, 1% Triton X-100, 0.1% SDS and 0.1% sodium deoxycholate), wash buffer B (50 mM Hepes pH 7.9, 500 mM NaCL, 1 mM EDTA, 1% Triton X-100, 0.1% SDS and 0.1% sodium deoxycholate), and wash buffer C (25 mM Tris–HCl pH 7.5, 1 mM EDTA, 250 mM LiCl, 0.5% NP-40 alternative and 0.5% sodium deoxycholate). Finally, the beads were washed with tris-EDTA Buffer for two times and eluted with elution buffer (10 mM Tris, pH 7.5, 1 mM EDTA, and 1% SDS) and incubated at 65 °C for 30 min. The eluted samples were treated with RNase A (Invitrogen, catalog no. EN0531) to a final concentration of 20 μg/ml and incubated at 65 °C for 8 h to digest any contaminated RNA. Next, the samples were treated with Proteinase K (Invitrogen, catalog no. 25530049) to a final concentration of 200 μg/ml and incubated at 45 °C for 2 h to digest proteins. Finally, the DNA was purified using a QIAquick PCR Purification Kit (QIAGEN, catalog. no. 28104) and used for qPCR analysis.

### RNA isolation, reverse transcription, and quantitative real-time PCR

The BRPF1 and ILF3 overexpressed in Huh-7 HCC cells, and total RNA was extracted using the TRIzol reagent (Invitrogen, catalog no. 15596026) according to the manufacturer’s protocol. The isolated RNA was quantified using spectrophotometer, and 1 μg RNA was treated with DNase I (1U/μl) (Invitrogen, catalog. no. EN0521). One microgram of total RNA and random hexamer primer was used to synthesize the cDNA using Verso cDNA Synthesis Kit (Invitrogen, catalog no. AB1453A) following the manufacturer’s protocol. Quantitative real-time PCR was performed in triplicate using the PowerUp SYBR Green Master Mix (Invitrogen, A25742) on a CFX96 touch real-time PCR detection system (Bio-Rad). Specific primers for each gene are collected from PrimerBank (55) for qPCR experiments (Table S6). All mRNA quantification data were normalized to GAPDH, which was used as an internal control.

## Data availability

All data are included in the manuscript and supporting information.

## Supporting information

This article contains supporting information. The supporting information contains peptide characterization including ESI-MS peptide data, HPLC purity traces for peptides, KEGG pathway analysis, Figs. S1–S22 and Tables S1–S6.

## Conflict of interest

The authors declare that they have no conflicts of interest with the contents of this article.
