# Supplementary material for: Uncovering the non-histone interactome of the BRPF1 bromodomain using site-specific azide-acetyllysine photochemistry
Source: J Biol Chem. 2023 Dec 10;300(1):105551. doi: 10.1016/j.jbc.2023.105551 (PMC10789646; doi:10.1016/j.jbc.2023.105551)
Supplement: Supporting Information [file mmc3.pdf]

Supporting Information

Uncovering the non-histone interactome of the BRPF1 bromodomain using site-specific azide-acetyllysine photochemistry

Soumen Barman,[1] Jyotirmayee Padhan,[1] and Babu Sudhamalla[1,*]

[1]Department of Biological Sciences, Indian Institute of Science Education and Research Kolkata, Mohanpur, West Bengal 741246, India

Table of Contents

1. Supplementary Tables S2
2. Supplementary Figures S3-S16

Supplementary Table S1: List of the genes used in the current study. The expression vector, antibiotic resistance and the affinity tag present for protein purification are provided.

Gene	Vector	Affinity tag	Resistance
BRPF1	pNIC28-Bsa4	N-6xHis	Kanamycin
M. j. TyrRS-tRNACUA pairs	pEVOL	None	Chloramphenicol
BRPF1	pcDNA6.2	V5	Ampicillin
ILF3	pcDNA3	Myc	Ampicillin

Supplementary Table S2: List of the forward primers designed for site-directed mutagenesis. Reverse primers used are the reverse-complement to the given forward primers.

BRPF1 Bromodomain mutants	Primer sequence
N651TAG	GAAGGACACAGGCTAGATCTTCAGCGAG
V662TAG	CGGTCCCTCTGTCTGAGTAGCCTGACTACCTAGACCAC

Supplementary Table S3: List of peptides **2-9** varying acetylation sites were synthesized and used for this study.

Peptide	Peptide Sequence	Molecular weight (Da)
H4(1-15)K5acK8ac - **2**	SGRG**Kac**GG**Kac**GLGKGGA	1370.51
H4(1-20)Kac4 (K5/K8/K12/K16) - **3**	SGRG**Kac**GG**Kac**GLG**Kac**GGA**Kac**RHRK	2160.44
H4(1-20)K12ac - **4**	SGRGKGGKGLG**Kac**GGAKRHRK-**TAMRA**	2171.47
H4(11-20)K16ac - **5**	GKGGA**Kac**RHRK-**TAMRA**	1548.88
H4(1-12)K5acK8ac - **6**	SGRG**Kac**GG**Kac**GLGK-**TAMRA**	1597.42
H4(1-20)Kac4 (K5/K8/K12/K16) - **7**	SGRG**Kac**GG**Kac**GLG**Kac**GGA**Kac**RHRK-**TAMRA**	2572.89
ILF3 (94-106)K100acK105ac - **8**	RVGLVA**Kac**GLLL**Kac**G	1407.74
ILF3(94-106)Kunac - **9**	RVGLVAKGLLLKG	1323.67

Supplementary Table S4: MALDI-MS characterization of wild-type BRPF1 and its mutants.

BRPF1 bromodomain	Calculated Mass (Da)	Observed Mass (Da) (MALDI-MS)
WT	16152.36	16153.3
N651AzF	16226.44	16227.4
V662AzF	16241.41	16242.4

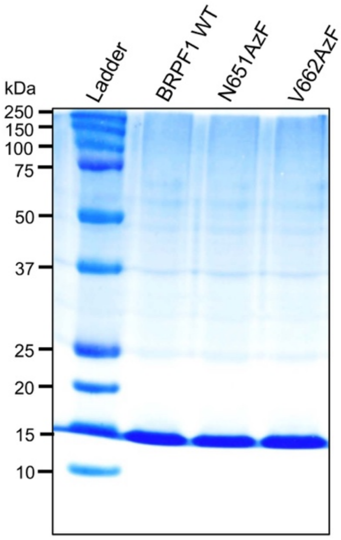

Supplementary Figure S1. Coomassie blue staining showing expression and purity of wild-type BRPF1 bromodomain and its mutants.

H4K5acK8ac (1-15)

HPLC REPORT

Sample:	Pep-460 SGRGK(ac)GGK(ac)GLGKGGA	Analyzed date: 21-03-2022
Analyst:	Dr.RS-SBio	
Column:	4.6×250mm,ChromCore 120 C18 5um	
Solvent A	A: 0.1% Trifluoroacetic Acid in 100% Acetonitrile	
Solvent B	B: 0.1% Trifluoroacetic Acid in 100% Water	

Gradient:		A	B
	0.0min	10%	90%
	25.0min	35%	65%
	25.1min	100%	0%
	30.0min	Stop	

Volume: 10μl

Wavelength: 220nm

Flow rate: 1.0ml/min

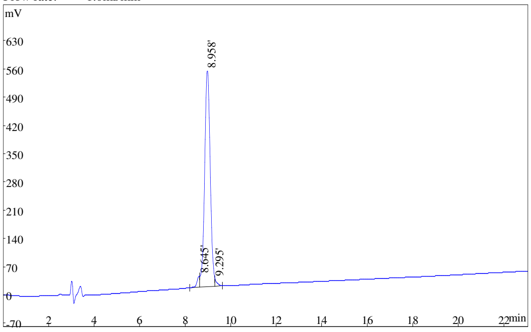

Rank	Time	Conc.	Area	Height
1	8.645	2.669	238653	27779
2	8.958	96.19	8601225	532768
3	9.295	1.145	102427	17006
Total		100	8942305	577553

Supplementary Figure S2. HPLC purity trace for the H4(1-15)K5acK8ac peptide **2**.

H4K5acK8ac (1-15)

MASS SPECTROMETRY REPORT

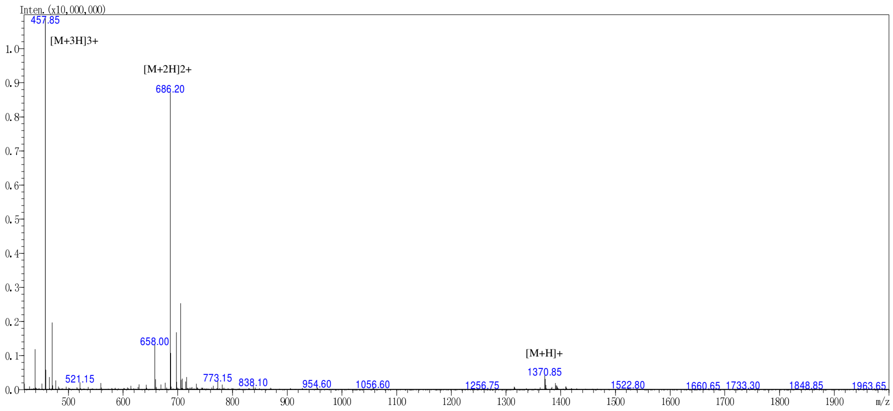

Sample Description		Instrument	SHIMADZU LCMS-2020		
Analyzed date:	22-03-2022	Probe:	ESI	Probe Bias:	+4.5kv
Analyst:	Dr.RS-SBio	Nebulizer Gas Flow:	1.5L/min	Detector:	1.2kv
Sample:	Pep-460 SGRGK(ac)GGK(ac)GLGKGGA	CDL:	-20.0v	T. Flow:	0.2ml/min
M.W.:	1370.51	CDL Temp.:	250 °C	B. Conc.:	50%H2O/50%ACN

Supplementary Figure S3. MS spectra for the H4(1-15)K5acK8ac peptide **2**.

HPLC REPORT
H4Kac4 (1-20)

Sample:	Pep-197 SGRGK(ac)GGK(ac)GLGK(ac)GGAK(ac)RHRK			Analyzed date: 11-12-2020
Analyst:	Dr.RS-SBio			
Column:	Gemini-NX 5µ C18 110A, 4.6*250mm			
Solvent A	A: 0.1% Trifluoroacetic Acid in 100% Acetonitrile			
Solvent B	B: 0.1% Trifluoroacetic Acid in 100% Water			
Gradient:		A	B	
	0.0min	10%	90%	
	25.0min	35%	65%	
	25.1min	100%	0%	
	30.0min	Stop		
Volume:	20µl			
Wavelength:	220nm			
Flow rate:	1.0ml/min			

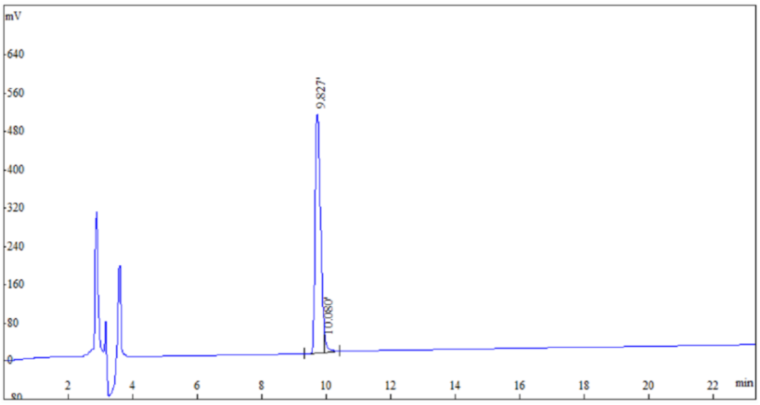

Rank	Time	Conc.	Area	Height
1	9.827	97.5303	5575313	499399
2	10.080	2.4697	141182	25521
Total		100	5716495	524920

Supplementary Figure S4. HPLC purity trace for the H4(1-20)Kac4 peptide **3**.

MASS SPECTROMETRY REPORT

H4Kac4 (1-20)

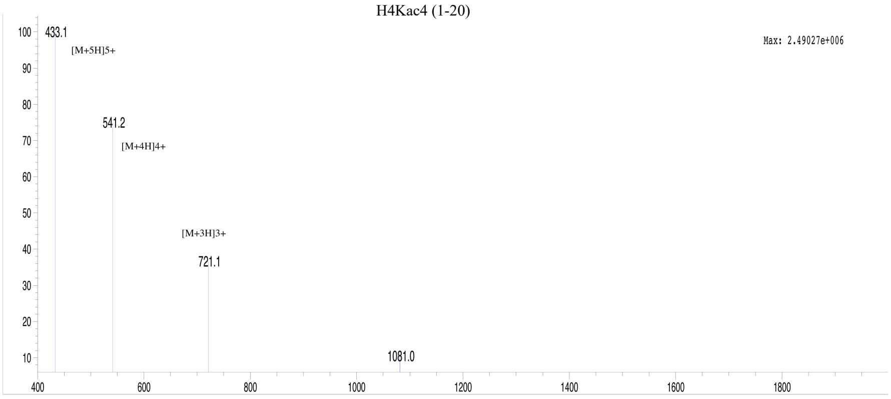

Sample Description		Instrument	Agilent-6125B		
Analyzed date:	12-12-2020	Probe:	ESI	Probe Bias:	4.5kv
Analyst:	Dr.AR-SBio	Nebulizer Gas Flow:	1.5L/min	Detector:	1.5kv
Sample:	Pep-197 SGRGK(ac)GGK(ac)GLGK(ac)GGAK(ac)RHRK	CDL:	-20.0v	T. Flow:	0.2ml/min
M.W.:	2160.44	CDL Temp.:	250 °C	B. Conc.:	50%H2O/50%ACN

Supplementary Figure S5. MS spectra for the H4(1-20)Kac4 peptide **3**.

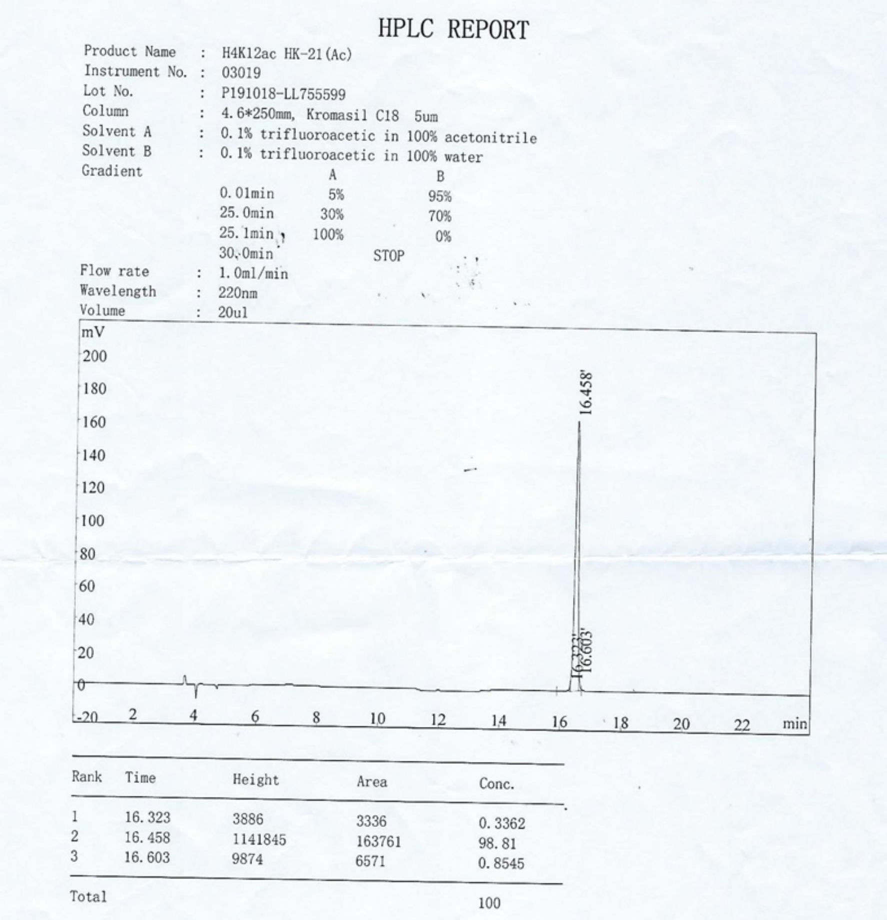

HPLC REPORT

Product Name : H4K12ac HK-21(Ac)
Instrument No. : 03019
Lot No. : P191018-LL755599
Column : 4.6*250mm, Kromasil C18 5um
Solvent A : 0.1% trifluoroacetic in 100% acetonitrile
Solvent B : 0.1% trifluoroacetic in 100% water
Gradient

	A	B
0.01min	5%	95%
25.0min	30%	70%
25.1min	100%	0%
30.0min	STOP	

Flow rate : 1.0ml/min
Wavelength : 220nm
Volume : 20ul

Rank	Time	Height	Area	Conc.
1	16.323	3886	3336	0.3362
2	16.458	1141845	163761	98.81
3	16.603	9874	6571	0.8545
Total				100

Supplementary Figure S6. HPLC purity trace for the H4(1-20)K12ac peptide **4**.

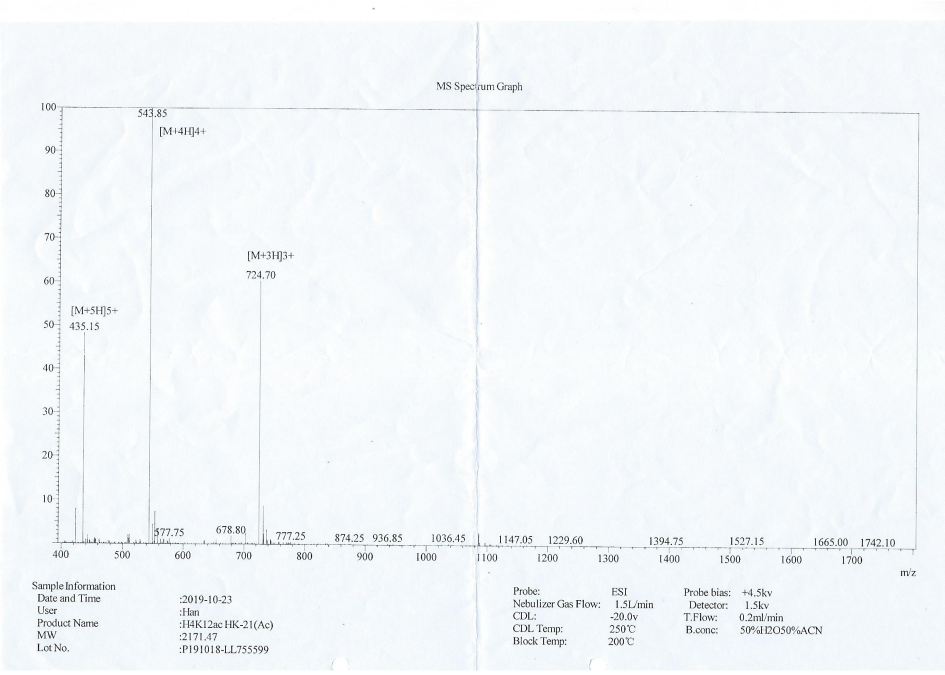

Supplementary Figure S7. Mass spectra for the H4(1-20)K12ac peptide **4**.

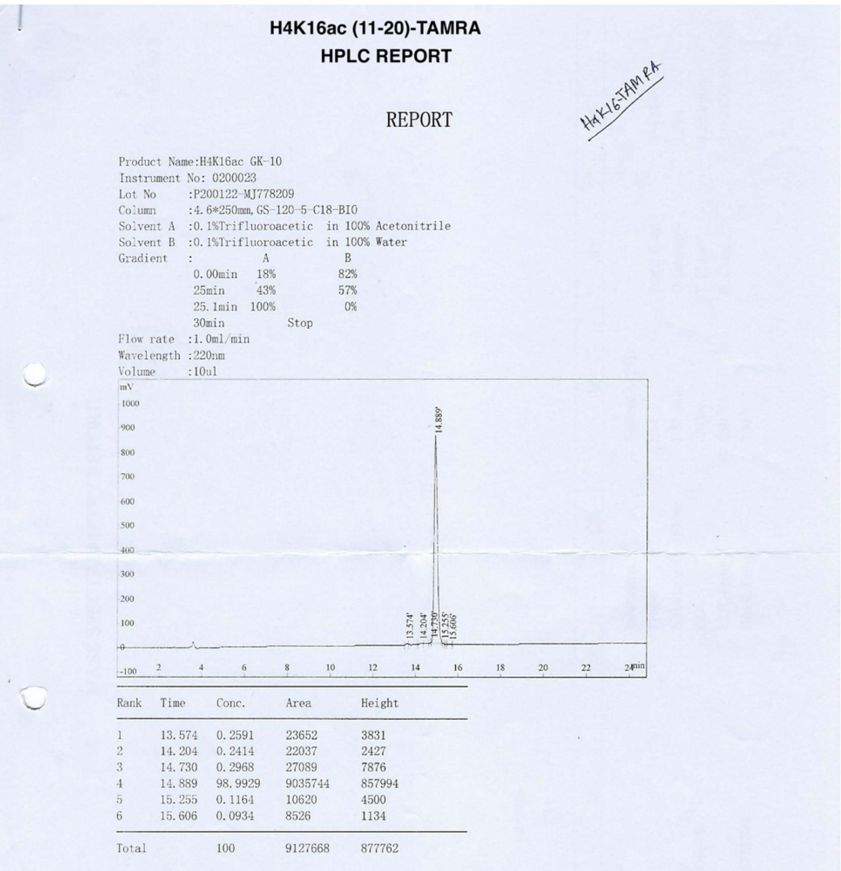

H4K16ac (11-20)-TAMRA
HPLC REPORT

REPORT

Product Name:H4K16ac GK-10
Instrument No: 0200023
Lot No :P200122-MJ778209
Column :4.6*250mm,GS-120-5-C18-BIO
Solvent A :0.1%Trifluoroacetic in 100% Acetonitrile
Solvent B :0.1%Trifluoroacetic in 100% Water

Gradient :	A	B
0.00min	18%	82%
25min	43%	57%
25.1min	100%	0%
30min	Stop	

Flow rate :1.0ml/min
Wavelength :220nm
Volume :10ul

Rank	Time	Conc.	Area	Height
1	13.574	0.2591	23652	3831
2	14.204	0.2414	22037	2427
3	14.730	0.2968	27089	7876
4	14.889	98.9929	9035744	857994
5	15.255	0.1164	10620	4500
6	15.606	0.0934	8526	1134
Total		100	9127668	877762

Supplementary Figure S8. HPLC purity trace for the H4(11-20)K16ac peptide **5**.

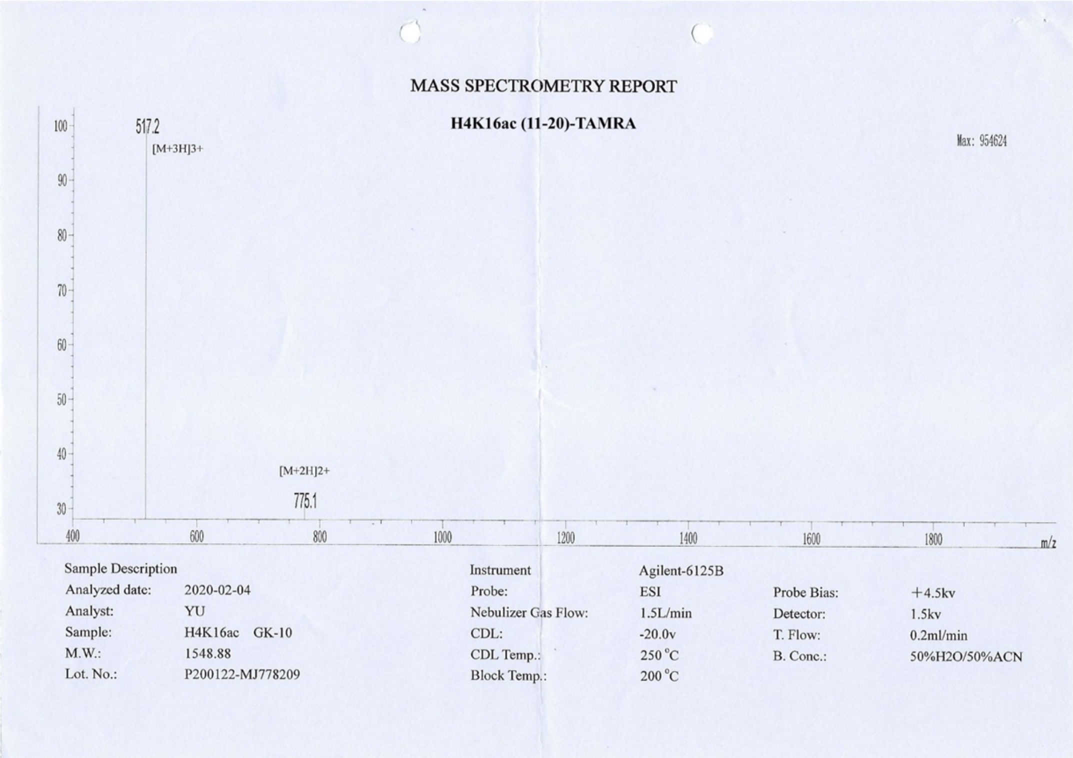

MASS SPECTROMETRY REPORT
H4K16ac (11-20)-TAMRA

Sample Description
Analyzed date: 2020-02-04
Analyst: YU
Sample: H4K16ac GK-10
M.W.: 1548.88
Lot. No.: P200122-MJ778209

Instrument: Agilent-6125B
Probe: ESI
Nebulizer Gas Flow: 1.5L/min
CDL: -20.0v
CDL Temp.: 250 °C
Block Temp.: 200 °C

Probe Bias: +4.5kv
Detector: 1.5kv
T. Flow: 0.2ml/min
B. Conc.: 50%H2O/50%ACN

Supplementary Figure S9. MS spectra for the H4(11-20)K16ac peptide **5**.

H4K5acK8ac (1-12)-TAMRA
HPLC REPORT

Sample:	Pep-168 SGRGK(ac)GGK(ac)GLGK-TAMRA		Analyzed date: 05-10-2020
Analyst:	Dr.RS-SBio		
Column:	4.6×250mm,Sinochrom ODS-BP 5μm		
Solvent A	A: 0.1% Trifluoroacetic Acid in 100% Acetonitrile		
Solvent B	B: 0.1% Trifluoroacetic Acid in 100% Water		

Gradient:

	A	B
0.0min	25%	75%
25.0min	50%	50%
25.1min	100%	0%
30.0min		Stop

Volume: 5μl
Wavelength: 220nm
Flow rate: 1.0ml/min

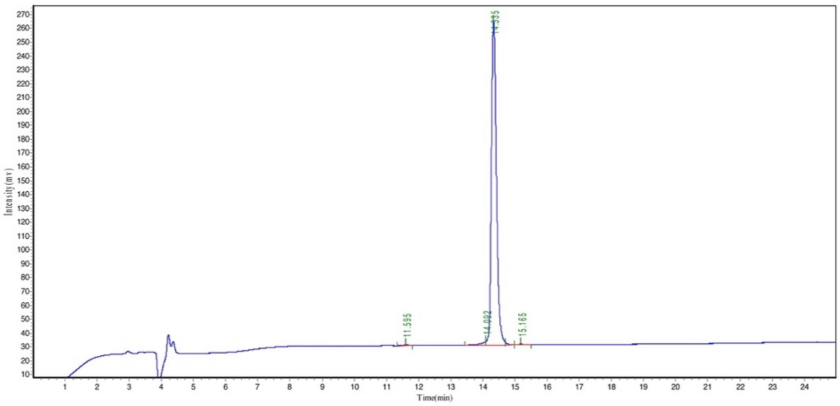

Peak	Time	Height	Area	Conc.
1	11.595	571.317	7500.777	0.2870
2	14.092	2403.828	24916.848	0.9532
3	14.335	233421.141	2566024.500	98.1669
4	14.335	2125.015	9224.838	0.3529
5	15.165	671.953	6272.946	0.2400
Total				100.000

Supplementary Figure S10. HPLC purity trace for the H4(1-12)K5acK8ac peptide **6**.

H4K5acK8ac (1-12)-TAMRA
MASS SPECTROMETRY REPORT

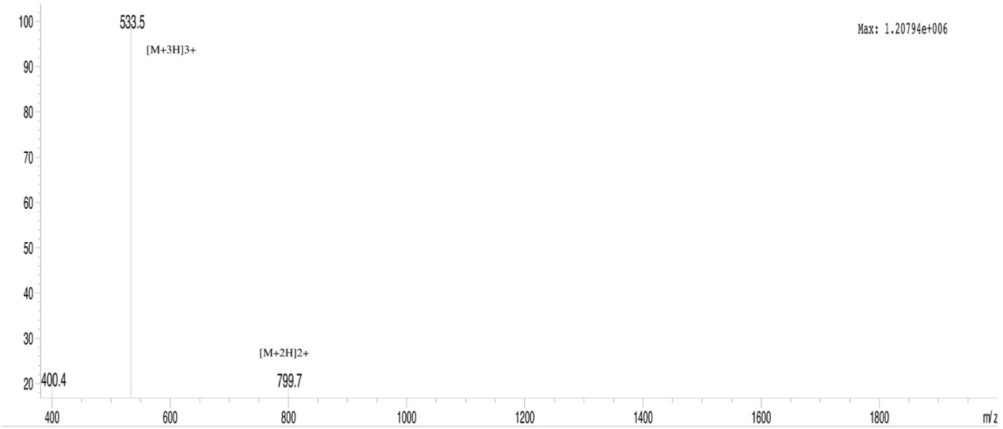

Sample Description		Instrument	Agilent-6125B		
Analyzed date:	06-10-2020	Probe:	ESI	Probe Bias:	4.5kv
Analyst:	Dr.AR-Sbio	Nebulizer Gas Flow:	1.5L/min	Detector:	1.5kv
Sample:	Pep-168 SGRGK(ac)GGK(ac)GLGK-TAMRA	CDL:	-20.0v	T. Flow:	0.2ml/min
M.W.:	1597.42	CDL Temp.:	250 °C	B. Conc.:	50%H2O/50%ACN

Supplementary Figure S11. Mass spectra for the H4(1-12)K5acK8ac peptide **6**.

H4K5acK8acK12acK16ac (1-20)-TAMRA

HPLC REPORT

Sample:	Pep-169 SGRGK(ac)GGK(ac)GLGK(ac)GGAK(ac)RHRK-TAMRA		Analyzed date: 08-11-2020
Analyst:	Dr.RS-SBio		
Column:	Symmetrix ODS-R, 4.6*250mm, 5µm		
Solvent A	A: 0.1% Trifluoroacetic Acid in 100% Acetonitrile		
Solvent B	B: 0.1% Trifluoroacetic Acid in 100% Water		
Gradient:		A	B
	0.0min	18%	82%
	25.0min	43%	57%
	25.1min	100%	0%
	30.0min	Stop	
Volume:	20µl		
Wavelength:	220nm		
Flow rate:	1.0ml/min		

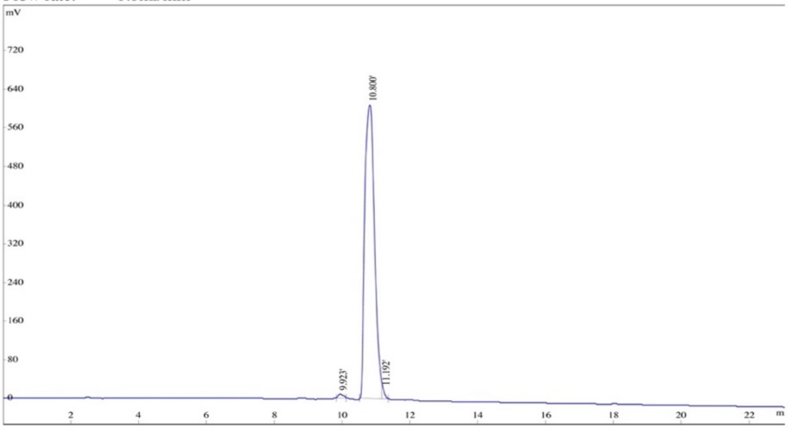

Rank	Time	Conc.	Area	Height
1	9.923	0.7119	83545	7885
2	10.800	98.4844	11557687	608544
3	11.192	0.8037	94316	21570
Total		100	11735548	637999

Supplementary Figure S12. HPLC purity trace for the H4(1-20)Kac4 peptide **7**.

H4K5acK8acK12acK16ac (1-20)-TAMRA

MASS SPECTROMETRY REPORT

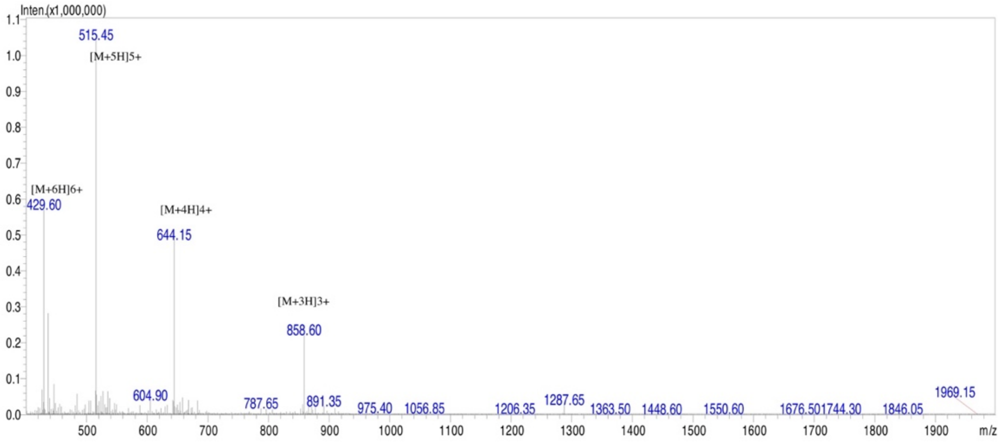

Sample Description		Instrument	SHIMADZU LCMS-2020		
Analyzed date:	09-11-2020	Probe:	ESI	Probe Bias:	4.5kv
Analyst:	Dr.AR-SBio	Nebulizer Gas Flow:	1.5L/min	Detector:	1.5kv
Sample:	Pep-169 SGRGK(ac)GGK(ac)GLGK(ac)GGAK(ac)RHRK-TAMRA	CDL:	-20.0v	T. Flow:	0.2ml/min
M.W.:	2572.89	CDL Temp.:	250 °C	B. Conc.:	50%H2O/50%ACN

Supplementary Figure S13. Mass spectra for the H4(1-20)Kac4 peptide **7**.

HPLC REPORT

Sample:	Pep-723 RVGLVA(Kac)GLLL(Kac)G	Analyzed date: 19-05-2023
Analyst:	Dr.RS-SBio	
Column:	4.6×250mm,Sinochrom ODS-BP 5μm	
Solvent A	A: 0.1% Trifluoroacetic Acid in 100% Acetonitrile	
Solvent B	B: 0.1% Trifluoroacetic Acid in 100% Water	

Gradient:

	A	B
0.0min	27%	73%
25.0min	52%	48%
25.1min	100%	0%
30.0min		Stop

Volume: 5μl
Wavelength: 220nm
Flow rate: 1.0ml/min

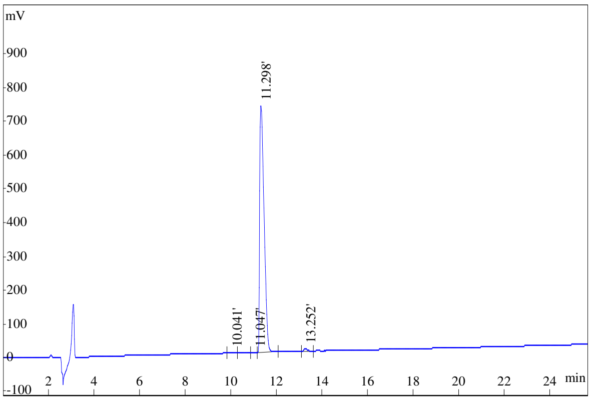

Rank	Time	Conc.	Area	Height
1	10.041	0.1751	15910	1988
2	11.047	0.09322	8472	1101
3	11.298	98.99	8996521	728024
4	13.252	0.7373	67007	6760
Total		100.0000		

Supplementary Figure S14. HPLC purity trace for the ILF3(94-106)K100acK105ac peptide **8**.

MASS SPECTROMETRY REPORT

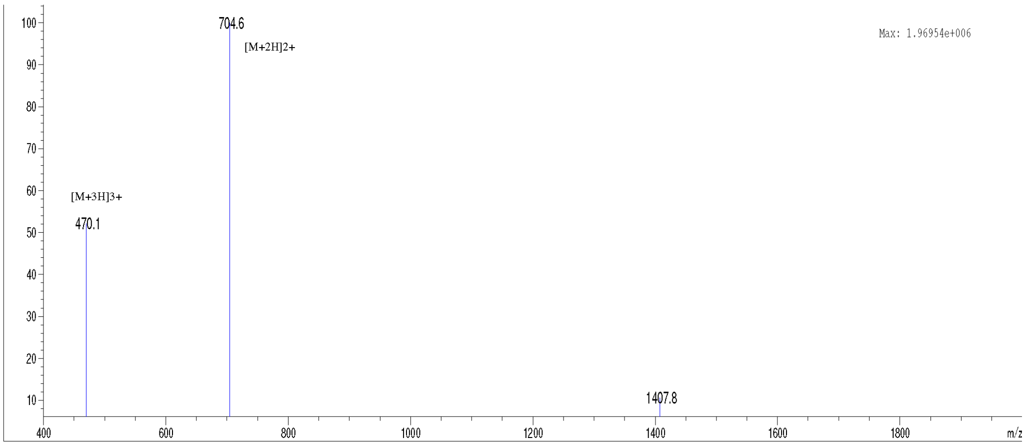

Sample Description		Instrument	Agilent-6125B		
Analyzed date:	20-05-2023	Probe:	ESI	Probe Bias:	+4.5kv
Analyst:	Dr.AR-SBio	Nebulizer Gas Flow:	1.5L/min	Detector:	1.5kv
Sample:	Pep-723 RVGLVA(Kac)GLLL(Kac)G	CDL:	-20.0v	T. Flow:	0.2ml/min
M.W.:	1407.74	CDL Temp.:	250 °C	B. Conc.:	50%H2O/50%ACN

Supplementary Figure S15. Mass spectra for the ILF3(94-106)K100acK105ac peptide **8**.

HPLC REPORT

Sample:	Pep-746 RVGLVAKGLLLKG	Analyzed date: 11-07-2023
Analyst:	Dr.RS-SBio	
Lot. No.:	P230608-SY1085333	
Column:	4.6×250mm,Sinochrom ODS-BP 5μm	
Solvent A	A: 0.1% Trifluoroacetic Acid in 100% Acetonitrile	
Solvent B	B: 0.1% Trifluoroacetic Acid in 100% Water	

Gradient:

	A	B
0.0min	19%	81%
25.0min	44%	56%
25.1min	100%	0%
30.0min		Stop

Volume: 5μl
Wavelength: 220nm
Flow rate: 1.0ml/min

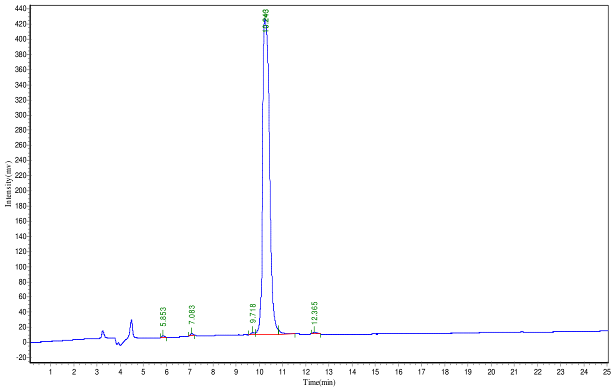

Peak	Time	Height	Area	Conc.
1	5.853	2749.410	21822.395	0.2516
2	7.083	2476.367	21740.799	0.2506
3	9.718	2824.157	27117.789	0.3126
4	10.243	416718.781	8524647.000	98.2714
5	10.725	6189.295	52931.246	0.6102
6	12.365	2262.278	26334.373	0.3036
Total				100.000

Supplementary Figure S16. HPLC purity trace for the ILF3(94-106) unacetylated peptide **9**.

MASS SPECTROMETRY REPORT

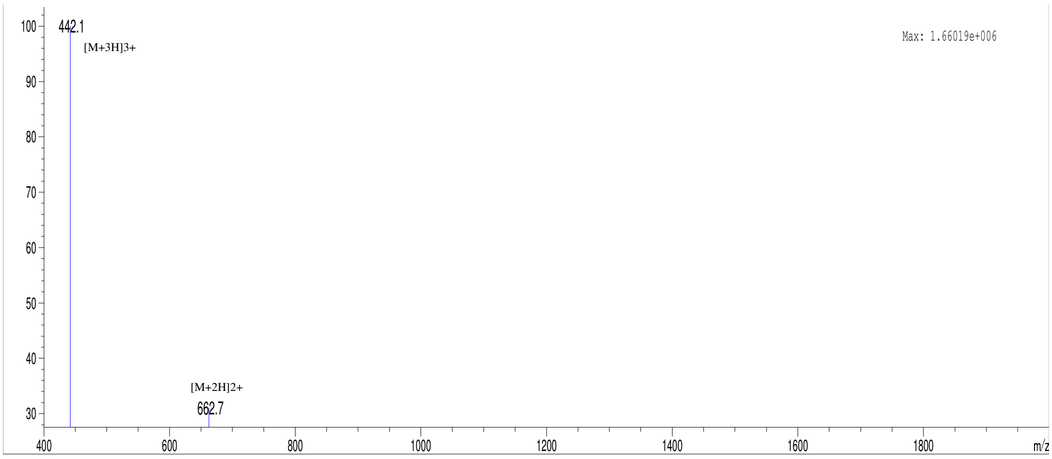

Sample Description		Instrument	Agilent-6125B		
Analyzed date:	12-07-2023	Probe:	ESI	Probe Bias:	+4.5kv
Analyst:	Dr.AR-SBio	Nebulizer Gas Flow:	1.5L/min	Detector:	1.5kv
Sample:	Pep-746 RVGLVAKGLLLKG	CDL:	-20.0v	T. Flow:	0.2ml/min
M.W.:	1323.67	CDL Temp.:	250 °C	B. Conc.:	50%H2O/50%ACN
Lot. No.:	P230608-SY1085333	Block Temp.:	200 °C		

Supplementary Figure S17. Mass spectra for the ILF3(94-106) unacetylated peptide **9**.

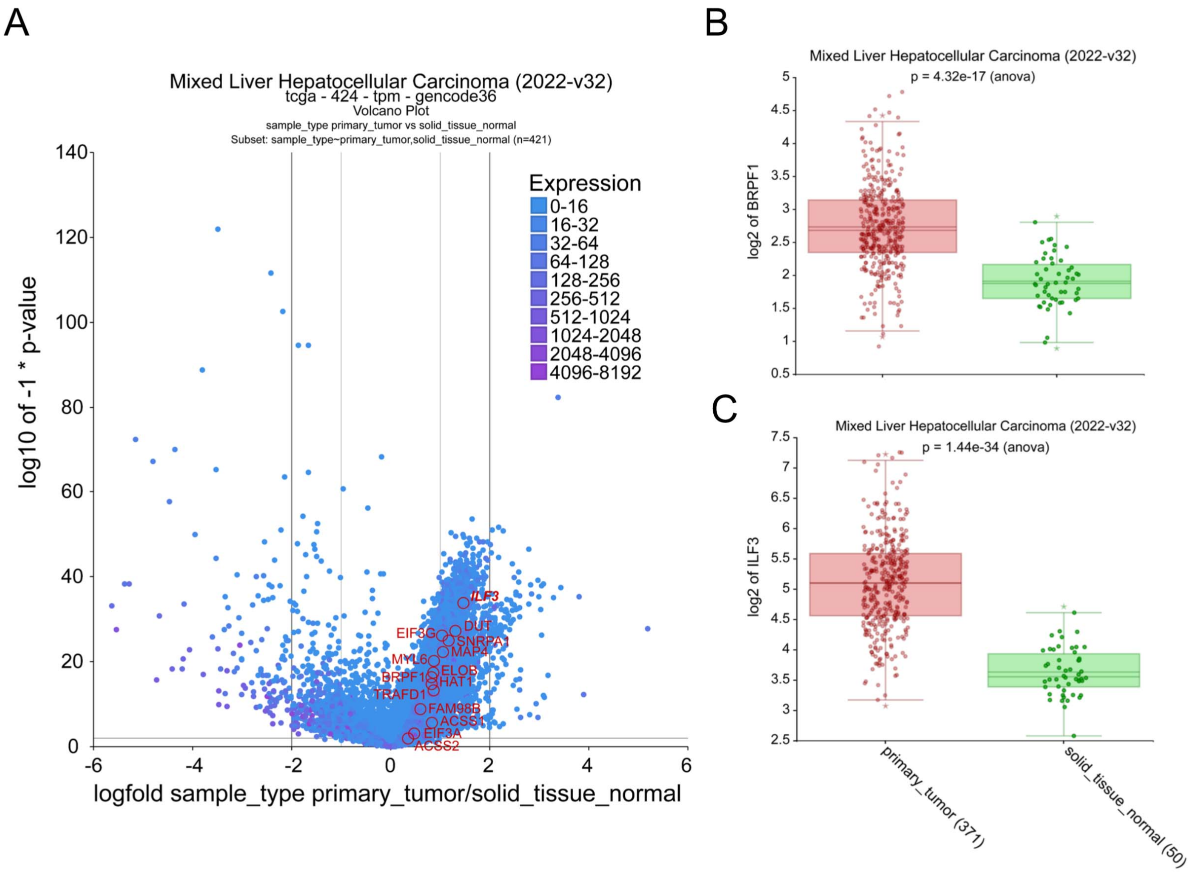

Supplementary Figure S18. Hepatocellular carcinoma (2022-v32) datasets analysis in R2: Genomics analysis and visualization platform (A) Volcano plot of tumor vs normal tissue. Each dot represents one gene and red circles are the genes that encode the proteins identified in the proteomics. (B) BRPF1 and (C) ILF3 gene expression of tumor vs normal tissue in HCC.

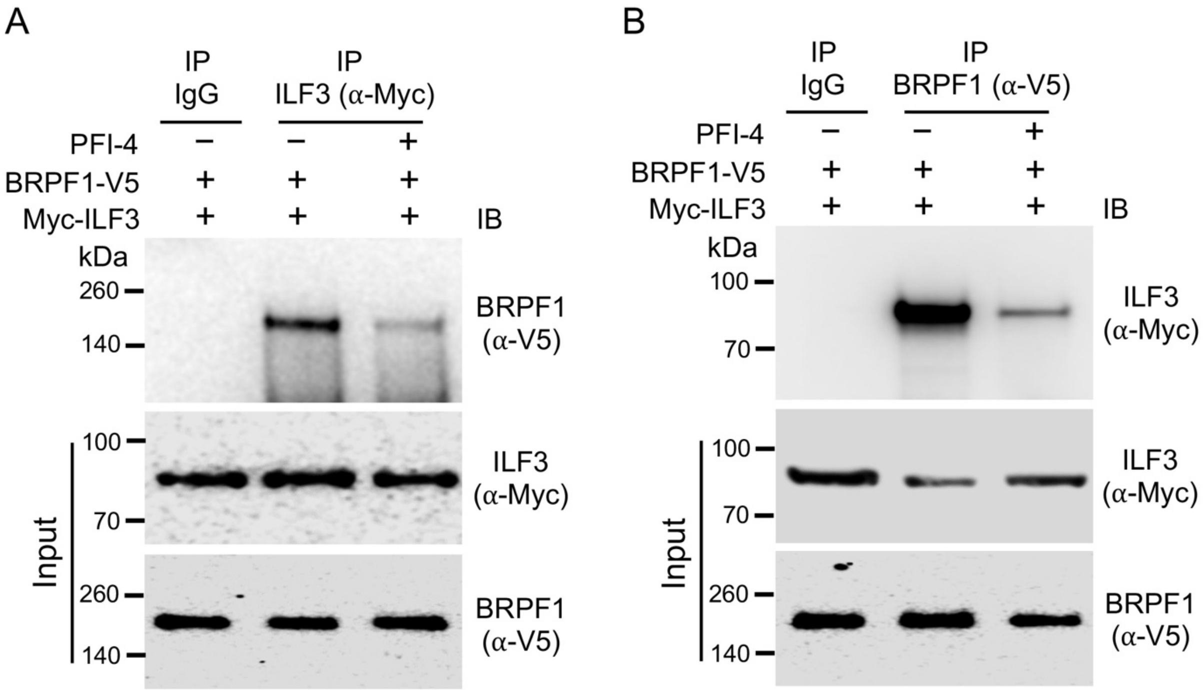

Supplementary Figure S19. Interaction of BRPF1 and ILF3. (A and B**)** Immunoprecipitation (IP) of BRPF1 and ILF3 in Huh-7 cells. The BRPF1 bromodomain specific inhibitor, PFI-4 significantly reduced the BRPF1-ILF3 interaction (uncropped western blot images of figure 7C and D).

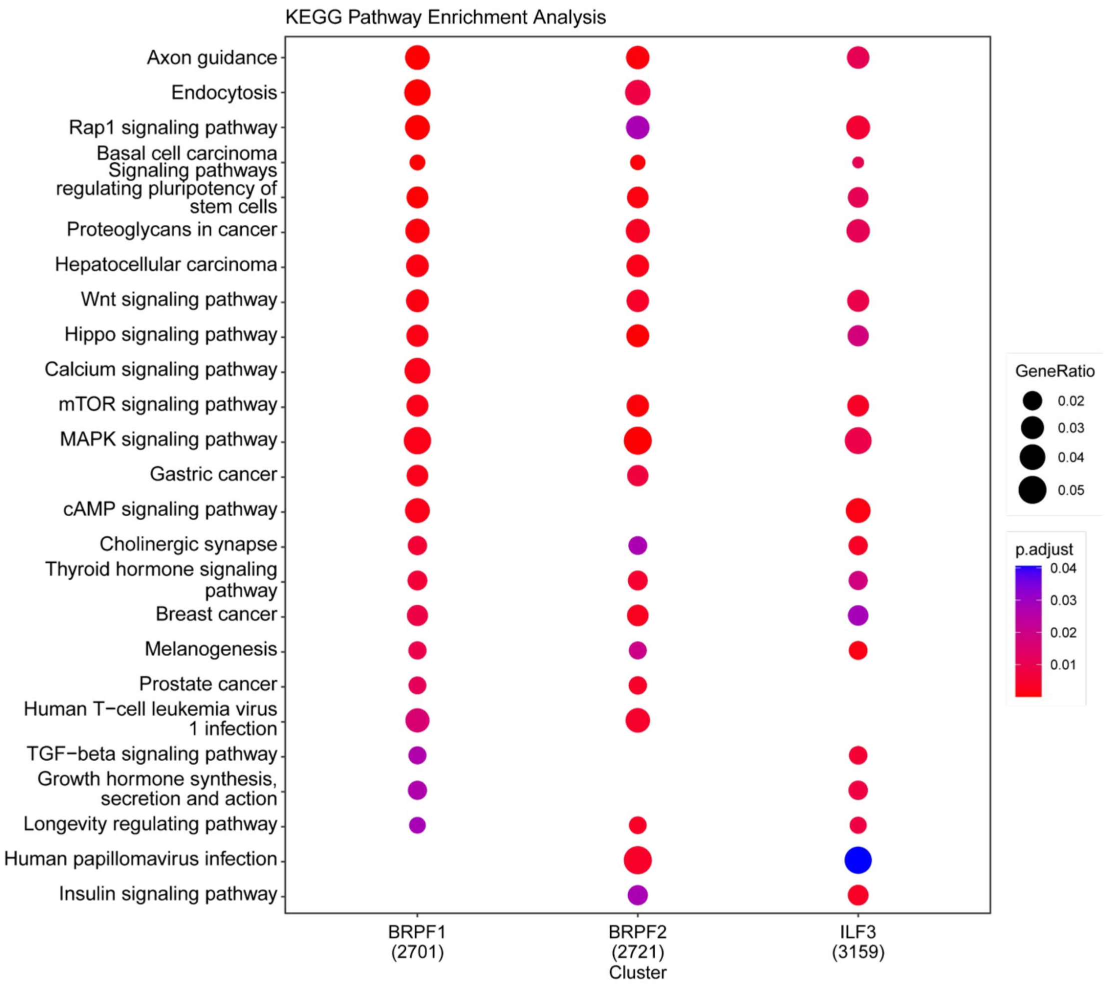

Supplementary Figure S20. The dot plot of the KEGG pathway enrichment analysis of the BRPF1, BRPF2, and ILF3. The area of each node represents the enriched gene ratio corresponding to BRPF1, BRPF2 and ILF3. The *p*-value is represented by a color scale; red represents high significance, while blue represents low. X-axis represents cluster of genes associated with functions represents in Y-axis.

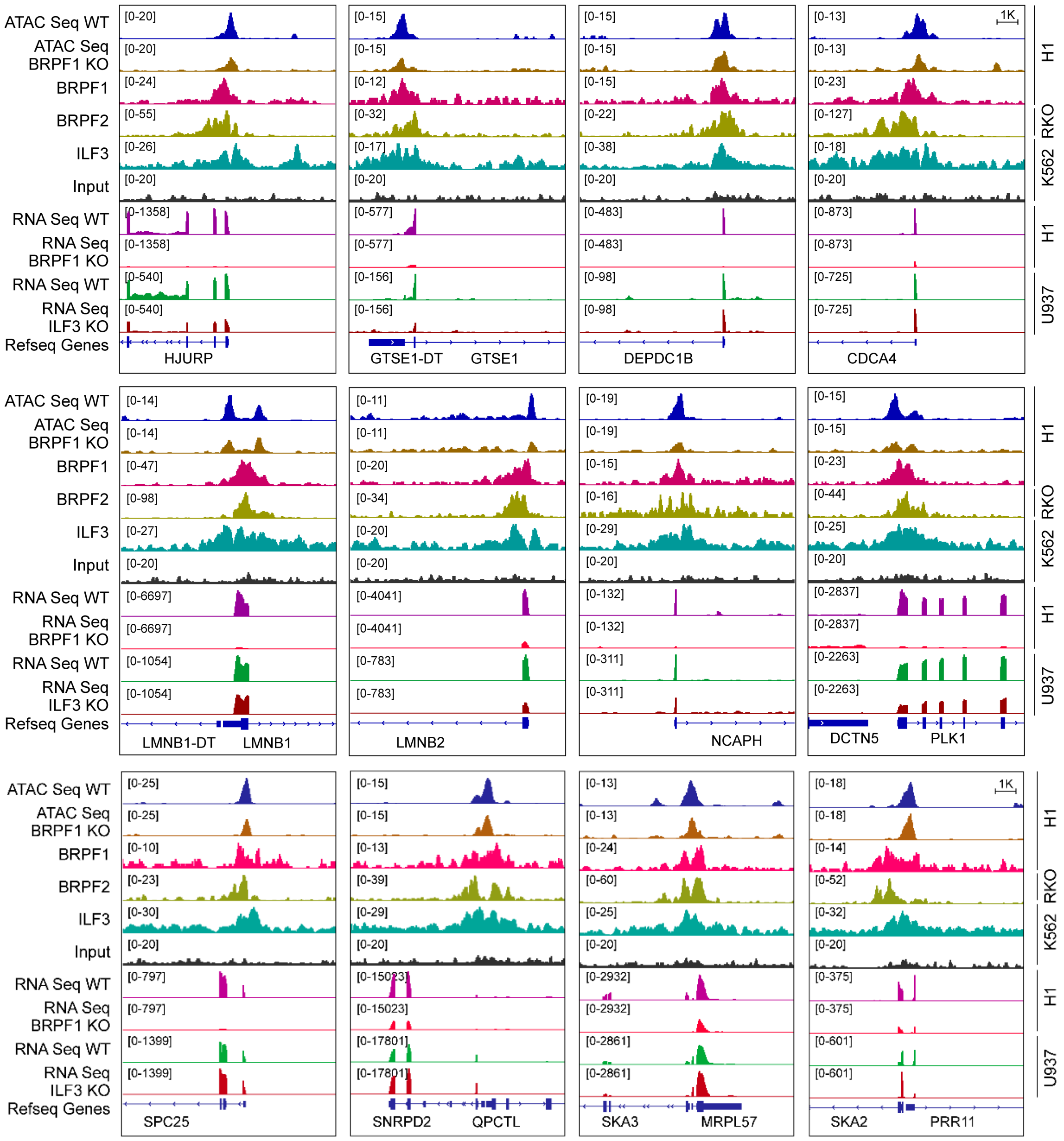

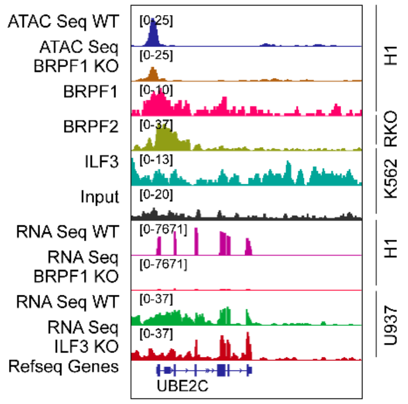

Supplementary Figure S21. Visualization of colocalization and expression of HCC hub genes. IGV snapshots of co-localization of BRPF1, BRPF2 and ILF3 (ChIP seq) along with chromatin accessibility (ATAC seq) and gene expression (RNA seq) profile of BRPF1 and ILF3 KO conditions at the promoter of HCC hub genes.

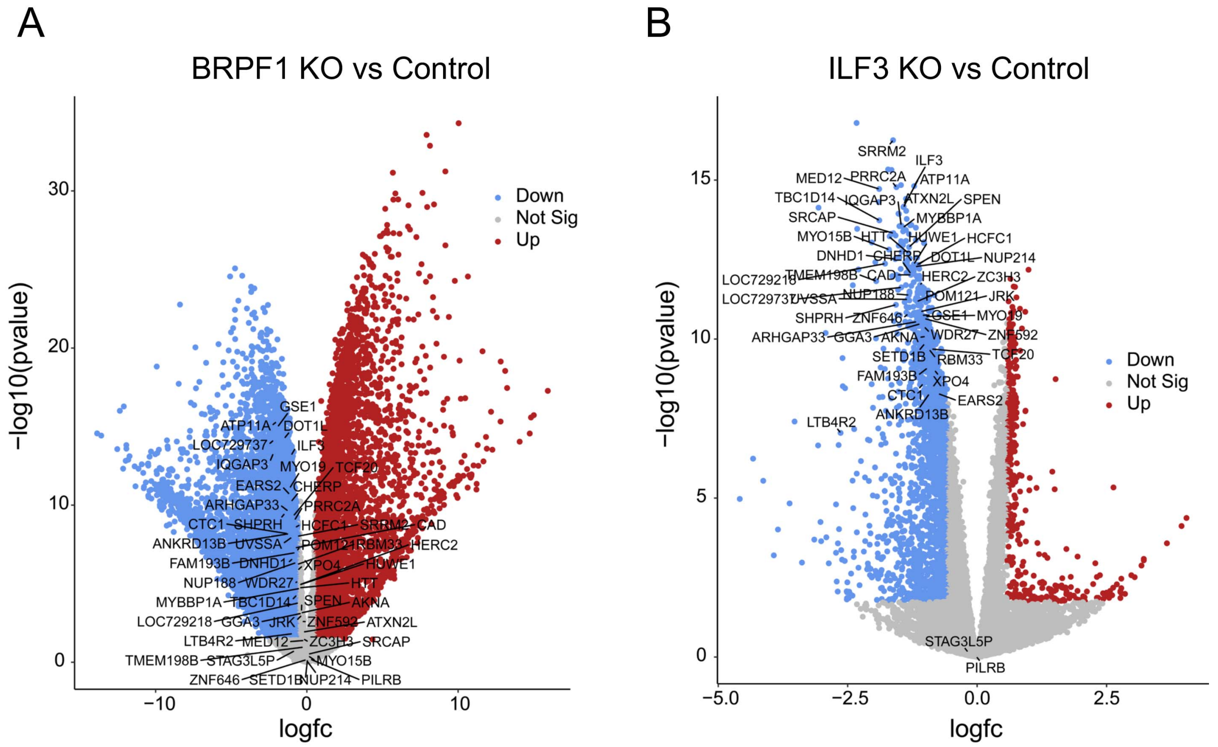

Supplementary Figure S22. Transcriptomic analysis. (A and B) Volcano plot. The logfc indicates the mean expression level for each gene. Each dot represents one gene. Grey dots represent no significant DEGs (*FC*>1.5, *p*<0.05) between KO group and control group, the blue dots represent down-regulated genes and red dots represent up-regulated genes.
